# Conduits of the Kremlin’s Informational Influence Abroad? How German-Language Alternative Media Outlets Are Connected to Russia’s Ruling Elites

**DOI:** 10.1177/19401612241230284

**Published:** 2024-02-20

**Authors:** Arista Beseler, Florian Toepfl

**Affiliations:** 1 University of Passau, Passau, Germany

**Keywords:** propaganda, alternative media, active measures, case study, Russia, influence

## Abstract

Extant research on alternative media in Western democracies has focused on scrutinizing their content, organization, production, and audiences. However, the extent to which alternative outlets are linked to powerful foreign actors has not yet been analyzed, despite the fact that a plethora of outlets have openly sided with Russia after its full-scale invasion of Ukraine in February 2022, spreading the Kremlin’s propagandistic narratives. To fill this gap, this study adopts a case study approach. It selects Germany as a revelatory case of a key target country of Russia’s foreign influence efforts, raising the question of how and to what extent German-language alternative media outlets are connected to Russia’s ruling elites. Grounded in qualitative analysis of a wide range of documents, this study proposes a categorization that divides the connections into three different types: organizational, media, and personal connections. Subsequently, it is demonstrated that half of the analyzed German-language alternative media outlets maintain at least one of these three types of connections to the Kremlin. These findings contribute to our knowledge of alternative media, as well as Russia’s overt and covert foreign influence operations, the so-called “active measures.” They also highlight the need for more transparency in alternative media landscapes in democratic contexts across the globe.

Since 2012, Jürgen Elsässer, the chief editor of the far-right German alternative media outlet *Compact*, has been organizing so-called *sovereignty conferences* in cooperation with the Institute of Democracy and Cooperation. This Kremlin-affiliated think tank based in France provided financial and organizational support for these events and is known, among other things, to have financially supported the French right-wing party *National Front*. In addition to this cooperation with a Kremlin-linked organization, Elsässer also frequently appears as a guest speaker on Russian state television and has, in the past, cooperated with the Russian foreign communication outlet RT (formerly *Russia Today*) German. This example illustrates the complexity of the ties that some central entities in the German-language alternative media landscape maintain with Kremlin-affiliated actors and organizations. However, to date, these connections have not been discussed in the academic literature. To fill this gap, this study raises the question of how and to what extent German-language alternative media outlets are connected to Russia’s ruling elites. By *Russia’s ruling elites*, or synonymously by the metaphor *the Kremlin*, we refer to Russia’s president Vladimir Putin and the small, tightly knit informal network of his closest allies, who make all key political decisions within Russia’s personalist authoritarian regime.

In Western democratic contexts, alternative media understand themselves—or are perceived—as a voice against the political and media mainstream (Holt et al. 2019). Germany, for instance, has a flourishing alternative media landscape, particularly when it comes to outlets with a right-wing political orientation (Heft et al. 2020; Müller and Freudenthaler 2022). In recent years, alternative media outlets have received much scholarly attention (Atkinson et al. 2022), for example, with regard to their dissemination of problematic content, such as dis- or misinformation about COVID-19 (Boberg et al. 2020; Frischlich et al. 2022). With most measures against the spread of COVID-19 having ceased in Europe, the war against Ukraine now takes precedence in the coverage of alternative media. Following Russia’s full-scale invasion of Ukraine in February 2022, there has been a shift to pro-Russian narratives. For example, pro-Kremlin narratives such as the alleged need for a “denazification” of Ukraine or disinformation about the Bucha massacre have featured prominently in a large number of German-language alternative media outlets (Amadeu Antonio Stiftung 2022).^1,2^

In the past, the Soviet Union has been known to employ so-called *active measures* to overtly and covertly exert influence in foreign countries to destabilize them and thus advance the Soviet Union’s position in the transnational political context (Abrams 2016; Rid 2020; Shultz and Godson 1984). These active measures ranged from open propaganda techniques, such as the use of foreign communication channels, to entirely covert tactics, such as the use of agents of influence, seemingly independent individuals who would use their positions to act in the Soviet Union’s favor (Abrams 2016; Rid 2020). However, active measures are not merely a thing of the past; various tactics have been updated in recent years by the Soviet Union’s most powerful successor state, Russia, to the digital age (Abrams 2016; DeBenedictis 2022; Rid 2020). A well-known example of the Kremlin’s digitalized active measures is the use of bots and paid human commenters (“trolls”) to influence public opinion in Western democracies, as was attempted in the 2016 U.S. presidential election (Rid 2020). Against this background, it appears plausible that the Kremlin might also reach out to web-based alternative media outlets in key target countries of its foreign communication efforts, seeking to influence, for instance, the coverage of Russia’s war against Ukraine.

However, no academic study has thus far analyzed these potential connections and their extent. In the literature on alternative media and active measures (see, e.g., Abrams 2016; Bradshaw et al. 2022; Elswah and Howard 2020; Klawier et al. 2022; Müller and Freudenthaler 2022; Schwarzenegger 2021), only state-sponsored outlets, such as RT and Sputnik, have been considered Kremlin-affiliated. To fill this research gap, we adopt a case study approach (Yin 2014) grounded in qualitative analysis of documents and investigative analysis of publicly available online information (Carter et al. 2021; Hayden 2019). The materials analyzed include reports from nongovernmental organizations, fact-checking services, newspaper articles, documents from government institutions, and information published by the alternative outlets themselves.

The connections scrutinized in this article have significant political implications and, from a normative standpoint, raise concerns for at least two reasons. First, although connections between media elites/institutions and political elites/institutions are observable in democratic settings, the connections examined in this study stand out due to their involvement with, and empowerment of, the ruling elites of a highly repressive, expansionist, and authoritarian regime. Notably, these elites are associated with Vladimir Putin, against whom the International Criminal Court issued an arrest warrant in March 2022 over allegations of war crimes. Second, it has been substantiated that Russia’s ruling elites have consistently endeavored to exert (covert) informational influence in democratic societies over the past decade, with the malign objective of destabilizing their constitutional orders (Decker 2021).

As the results of our analysis show, approximately half of the German-language alternative media outlets maintain connections with Russia’s ruling elites or affiliated actors at the organizational, media, or personal levels. In the following sections, we first summarize the current literature on alternative media and active measures before presenting and discussing the findings of this case study.

## Alternative Media Landscapes: Informal Connections With Foreign Actors Disregarded

Drawing on the work of Holt et al. (2019), this paper defines alternative media as “a self-perceived corrective of ‘traditional’, ‘legacy’ or ‘mainstream’ news media in a given sociocultural and historical context” (862). This relational, non-normative approach establishes the difference between mainstream and alternative outlets on a spectrum rather than as a fixed dichotomy. According to this definition, an outlet falls somewhere on the spectrum of *alternativeness* as soon as it takes an approach contrary to the current mainstream position, considering the outlet’s self-proclaimed orientation. The characteristic of *alternativeness* may manifest itself across different dimensions, namely the producer, content, organizational, and system levels (Holt et al. 2019: 863; see also Sandoval and Fuchs 2010 for a similar approach). Thus, alternative media may distinguish themselves by (1) producing content in non-conventional ways as well as by non-conventional producers (e.g., participatory journalism; Holt et al. 2019; Ihlebæk et al. 2022), (2) creating non-conventional content (such as non-mainstream topics or opinions; see, e.g., Buyens and Van Aelst 2021; Freudenthaler and Wessler 2022; Heft et al. 2020; Klawier et al. 2022; Müller and Freudenthaler 2022), (3) relying on non-conventional organizational structures (such as one-person outlets or non-conventional hierarchies; see, e.g., Aslan Ozgul and Veneti 2022; Harcup 2005; Heft et al. 2020; Ihlebæk et al. 2022; Issawi 2021), or (4) by serving a non-conventional systemic purpose (such as being the voice of the political opposition in repressive contexts; see, e.g., Issawi 2021; Leung and Lee 2014).

In recent years, a plethora of academic literature has focused on these various aspects of alternative media, as well as on its audiences (e.g., Noppari et al. 2019; Schulze 2020; Schwarzenegger 2022). The focus has been on right-wing alternative media, particularly in the German (e.g., Klawier et al. 2022; Müller and Freudenthaler 2022) and Scandinavian (e.g., Ihlebæk and Nygaard 2021; Mayerhöffer 2021) contexts, due to the rise of (far-)right hyper-partisan outlets (Haller and Holt 2019; Heft et al. 2020; Müller and Freudenthaler 2022). While scholars caution against generalizing alternative media as purely antagonistic and emphasize that it is “paramount to acknowledge and understand the diversity and range of alternative media” (Schwarzenegger 2022: 2), it can be said that radical or anti-democratic actors exist within the diverse sphere of alternative media and that, depending on their content and reach, they may weaken democracies (Ihlebæk et al. 2022: 1278).

The relations between alternative media and foreign actors have been discussed in the literature; however, it has almost exclusively been with a focus on Russia’s two major official foreign communication outlets, RT and Sputnik. As the key goals of these officially Kremlin-affiliated outlets, the literature commonly highlights sowing distrust in Western governments and institutions, thus destabilizing democracies (Elswah and Howard 2020; Wagnsson 2022). RT and Sputnik are often included in research on alternative media, highlighting their affiliation with the Kremlin (Bachl 2018; Bradshaw et al. 2022; Frischlich et al. 2022; Ihlebæk et al. 2022; Klawier et al. 2022; Müller and Freudenthaler 2022; Schwarzenegger 2021; Schweiger 2017). However, even after the major dissemination channels of RT and Sputnik have been blocked in Europe following Russia’s full-scale invasion of Ukraine, the Kremlin’s narratives still feature prominently in Germany’s alternative media outlets.^3,4^

In the wake of Russia’s full-scale invasion, additional voices disseminating pro-Kremlin content established themselves on various social media platforms, not only in Germany but also in several other European countries including Spain and Italy.^5,6^ While the newly created outlets are commonly referred to as (political) *influencers*, they can also be considered alternative media. As Ihlebæk et al. (2022) noted, alternative media can take many forms, including “one-person initiatives” (1273). Thus, in this study, we understand influencers as a subtype of alternative media as long as they (1) focus on timely or political topics, similar to conventional media, (2) consider themselves in opposition to the mainstream media landscape, and (3) present themselves as a kind of media brand (see Bause 2021 for a similar approach).

In the extant academic literature, no systematically collected knowledge is available about the organizational background of German-language alternative media outlets, let alone their potential connections to foreign actors. To fill these gaps, this study aims to empirically investigate the connections between German-language alternative media outlets and the Kremlin. Therefore, we pose the following research question: *How, and to what extent, are leading German-language alternative media outlets connected to Russia’s ruling elites?*

## Active Measures: How Russia Updated Soviet Practices to the Digital Age

The Soviet Union, and Russia as its most powerful successor state, have a history of conducting overt and covert operations with the goal of exerting influence and furthering Russia’s standing in the (geo-)political sphere, going back as early as the beginning of the twentieth century with the Bolsheviks’ effort to abolish the Russian Tsarist empire (Kux 1985; Lasswell 1951; Shultz and Godson 1984). Such tactics were especially prevalent during the Cold War, when the term *active measures* was used by Soviet intelligence agencies to refer to “a wide span of practices including disinformation operations, political influence efforts, and the activities of Soviet front groups and foreign communist parties” (Kux 1985: 19) with the “common goal of enhancing Soviet influence, usually by tarnishing the image of opponents” (Kux 1985: 19).

These tactics can be divided into three broader categories: white, gray, and black active measures (Abrams 2016; Kux 1985). While *white active measures* describe overt activities, such as official Russian media outlets or open engagement of diplomatic tools or institutions, *gray active measures* refer to more hidden tactics, such as funding “front organizations” (ostensibly independent organizations that were controlled by the Soviet leadership) or cultivating connections to similarly aligned political parties in other countries (Abrams 2016; Kux 1985). In the media field, the Soviet Union sponsored the “International Organization for Journalists” as a major front organization (Kux 1985: 22). Moreover, reminiscent of what is described in this article, both the Tsarist Empire and the Soviet Union provided covert subsidies to selected ideologically aligned journals (Kux 1985). Lastly, *black active measures* describe entirely covert tactics, for example, forging and disseminating false information or utilizing agents of influence (Abrams 2016; Kux 1985). Agents of influence are entities who use their “position, influence, power, and credibility to promote the objectives of a foreign power in ways unattributed to that power” (Shultz and Godson 1984: 38) in order to secretly distribute regime-aligned propaganda (DeBenedictis 2022).

Although such tactics are mostly discussed in a historical context, Russia has never completely ceased to implement active measures after the end of the Cold War (DeBenedictis 2022; Rid 2020). At the time of this writing, for example, the Russian news agency *TASS* was tightly controlled and supervised by the Kremlin (Watanabe 2017), and some European political parties, such as France’s *National Front*, Italy’s *Lega Nord*, as well as Germany’s *Die Linke* and *Alternative für Deutschland* (AfD, English: *Alternative for Germany*), continued to maintain (often intransparent) connections to Kremlin-aligned politicians or organizations (Fisher 2021). Moreover, Russia adapted certain active measures to the digital age. The internet became a viable “battleground” for Russia’s intelligence agencies, who use tactics such as hacking computer systems, leaking sensitive information, conducting large-scale trolling, and using bots to spread pro-Kremlin narratives (Abrams 2016; DeBenedictis 2022; Rid 2020). A notorious example of such tactics is the 2016 U.S. presidential election, where the Kremlin-affiliated *Internet Research Agency* (IRA) sought to sway the opinion climate on U.S.-based social networks in Trump’s favor by, among others, spreading disinformation (Ehrett et al. 2022; Helmus 2018: 19).

Another facet of contemporary Russia’s active measures is state-sponsored foreign communication outlets such as RT and Sputnik (Abrams 2016). These outlets have often been referred to as the Kremlin’s mouthpieces, tools of soft power or even information warfare, mediators of mis- and disinformation, such as conspiracy theories, as well as distributors of anti-Semitic and Islamophobic content (for overviews of the literature on RT and Sputnik, see Crilley et al. 2020; Elswah and Howard 2020; Wagnsson 2022). However, as Abrams (2016) argued, “RT and other state-controlled media outlets represent only one facet of a much larger influence campaign—a single tool in a range of understudied activities that constitute a concerning gap in the West’s broader ‘soft-containment’ of Putin’s Russia” (6). After Russia’s full-scale invasion of Ukraine, RT’s and Sputnik’s content was blocked on many platforms across Europe. Experts have argued that RT and Sputnik, as former key distributors of the Kremlin’s narratives to foreign audiences, might be substituted by alternative media outlets connected informally and covertly to the Kremlin.^6^ From the lens of the literature on active measures, such Kremlin-aligned alternative media outlets might—unwittingly or wittingly—serve Russia’s ruling elites by covertly exerting influence in foreign countries. Thus, the instrumentalization of foreign alternative media outlets might be another asset of the Kremlin’s active measure toolbox.

## Methodological Approach

### Case Selection: The German-Language Alternative Media Landscape

To analyze the connections between German-language alternative media outlets and Russia’s ruling elites, we adopted a case study approach. The case study approach describes “an empirical inquiry that investigates a contemporary phenomenon (the ‘case’) in depth and within its real-world context” (Yin 2014: 16). In this article, we consider the German-language alternative media landscape as a “revelatory case” (Yin 2014: 52). That is, we have chosen this case because it allows us to examine a phenomenon that has been “previously inaccessible [or not subject] to social science inquiry” (Yin 2014: 52). We undertake this analysis because we expect that some of our findings and the concepts proposed (e.g., our tripartite typology of conduits of influence) lend themselves to “analytical generalization” (Yin 2014: 68) to the alternative media landscapes of neighboring European countries and other democratic contexts around the globe, which are targeted by the Kremlin’s foreign propaganda efforts. At the same time, we acknowledge that our case has some particularities.

As one of the most influential (politically and economically) countries in Europe, Germany has long been a key target of Russian information influence (Decker 2021). It can therefore be assumed that the Kremlin’s propaganda efforts are particularly intense, visible, and traceable in this context. Moreover, in the decades before Russia’s full-scale invasion of Ukraine, Germany had relatively friendly and close relations with Russia. Although East Germany was a satellite state of the Soviet Union and under Moscow’s control for more than 40 years (1949–1990), many German citizens, organizations, and parties, such as the social-democratic political party *SPD*, have long had good relations with Russia. Finally, it needs to be mentioned that our case analysis is not limited to outlets located in Germany but considers all German-language outlets, making Austrian or Swiss outlets relevant as well. This decision was based on the study of Heft et al. (2020: 31), who found that Austria-based alternative media outlets attract relatively large numbers of German users.

#### Selection of Outlets: The twenty Most Popular German-Language Alternative Outlets

To select outlets for analysis, we proceeded in three steps. First, we created a comprehensive list of German-language alternative media outlets by drawing on the literature on German-language alternative media (Bachl 2018; Boberg et al. 2020; Heft et al. 2020; Klawier et al. 2022; Müller and Freudenthaler 2022; Müller and Schulz 2021; Schulze 2020; Schwaiger 2022; Schwarzenegger 2022; Schweiger 2017), as well as on additional exploration and snowball sampling, scrutinizing the linking and mentioning behavior of the outlets. As justified in the previous section, we added political influencers to our list if they portrayed themselves as media brands opposed to the mainstream media landscape. After compiling a list featuring a total of fifty relevant outlets, we collected information on these outlets’ key user metrics, including monthly website visits (sourced from SimilarWeb), as well as follower counts on Facebook, Twitter, Instagram, Telegram, and YouTube. To focus the analysis on the most popular German-language alternative media outlets, we stipulated a threshold of at least one million website visitors per month or at least 100,000 followers on any given social media platform. This resulted in our final list of the twenty most popular outlets (see Table 1). To contextualize the findings of this article, the user metrics and follower numbers of these twenty alternative media outlets need to be put into perspective and contrasted with those of Germany’s legacy news media. To briefly mention but two examples, the website of the news program of Germany’s public broadcaster ARD, *Tagesschau.de*, as of 2023, received approximately 116 million monthly website visits, while its YouTube channel had 1.3 million subscribers (all metrics in this paragraph were retrieved in August 2023 from SimilarWeb). *BILD*, Germany’s biggest-selling daily tabloid newspaper, received around 215 million visits to its website and had approximately 1.5 million followers on YouTube.

**Table 1.** The twenty Most Popular German-Language Alternative Outlets as of June 2022.

Outlet name	Website	Facebook	Twitter	Instagram	Telegram	YouTube
Tichys Einblick	4.9M	64,264	291,700	NA	10,569	136,000
Reitschuster	4.5M	100,319	132,700	75,700	282,000	353,000
Achse des Guten	4.3M	52,039	63,000	1,320	7,630	111,000
Epoch Times	3.4M	876,455	14,200	11,100	42,173	16,500
PI-News	3.1M	NA	4,643	NA	5,220	NA
Journalistenwatch	2.5M	NA	NA	NA	121,000	1,800
Anti-Spiegel	2.2M	8,419	17,400	482	69,330	87,300
Report24	2.2M	NA	NA	NA	38,611	428
NachDenkSeiten	2.1M	99,945	40,400	NA	11,914	82,000
Wochenblick	1.9M	79,287	NA	2,795	59,885	6,960
Junge Freiheit	1.7M	129,663	55,700	18,000	5,328	64,600
MMnews	1.1M	4,767	3,369	74	41,100	116,000
DWN	1.0M	93,939	6,708	751	569	NA
Apolut/KenFM	897K	2,848	NA	7,023	116,000	NA
AUF1.tv	842K	5,967	NA	6,400	197,481	NA
Compact	593K	NA	32,800	NA	60,175	151,000
Kla.tv	434K	7,154	NA	NA	56,668	121,000
Neues aus Russland	20.7K	NA	NA	5,195	146,994	NA
eingeSCHENKt.TV	17.9K	33,176	1,879	2,499	22,252	116,000
Alles Ausser Mainstream	NA	NA	4,599	3,171	162,090	92,700

*Note*. All user metrics were collected on Jun. 14, 2022. Website metrics refer to monthly visits as provided by similarweb.com, social network metrics to followers or subscriptions.

#### Data Collection

For all outlets included in Table 1, we collected a wide range of documents containing information about their (presumed) connections to Russia’s ruling elites. The materials collected included news articles, fact-checking and think tank reports, and other publicly available online content. An innovative investigative open-source intelligence approach (OSINT approach; see Carter et al. 2021; Hayden 2019) to data collection and analysis allowed for a broad collection of information from multiple platforms, databases, and sources, with the goal of critically comparing claims by different sources and triangulating knowledge on the topic at hand. Adopting this approach, we also scrutinized the information provided by the analyzed alternative media outlets themselves, their authors, or related outlets. These arguably less reputable sources were interpreted in context and primarily consulted for first-hand accounts (such as attendance at an event or cooperation with other outlets). The documents were collected using various library databases for trustworthy (journalistic) content of quality media, mostly based in Germany or Austria. Additionally, we searched specific, thematically relevant websites for information, such as fact-checking services or media analysis projects. The search queries used ranged from “[*name of alternative media outlet or its chief editor*]” to “[*name of alternative media outlet*] AND russia OR putin OR kremlin.” The following section presents our findings with reference to 191 key documents, which we identified as containing particularly relevant or dense information on the selected alternative media outlets and their connections to the Kremlin. We provide a comprehensive list of these source documents in the Supplemental Information file. A case study database containing all source documents is available upon request.

#### Data Analysis

We coded the 191 key documents qualitatively using MAXQDA, following the suggestions of Kuckartz (2019). We opted to use a combination of concept-driven and data-driven coding: first, broad conceptual codes were derived (in our case, the relevant alternative media outlets), and second, types of connections were then derived based on the analyzed documents. This approach combines the advantages of deductive and inductive coding, providing a broad coding frame in the beginning, which is then refined during the subsequent coding process. While coding, the reputability of the source material was assessed, and information was only considered valid if two reputable sources provided the same information. The analysis was case-oriented, allowing us to “identify similarities between cases, identify extreme cases, and form types” (Kuckartz 2019: 187). After the coding process, we summarized the findings for each alternative media outlet and then developed a typology of connection types.

## Findings

### Types of Connections Between Alternative Media Outlets and Russia’s Ruling Elites

To systematize the various ways in which alternative media and Russia’s ruling elites are connected (implicitly or explicitly), we divided the connections observed into three different categories: organizational, media, and personal connections.

#### Organizational Connections

This overarching category includes all connections between outlets and Kremlin-affiliated organizations or institutions. It entails officially cooperating with an organization connected to Russia’s ruling elites, which may include direct financial support or sponsorships, hosting joint public events, such as conferences, and meetings in official contexts between representatives of the outlets and representatives of Russia’s ruling elites, such as politicians or oligarchs.

A prime example of official cooperation with Kremlin-affiliated organizations or institutions is epitomized by the right-wing German-language alternative media outlet *Compact*, led by Jürgen Elsässer. As such, Compact serves as an official partner of the German Centre for Continental Cooperation, a Kremlin-affiliated organization (D22, D27).^7^ In addition, Compact and a French Kremlin-affiliated think tank located in Paris, the Institute of Democracy and Cooperation, cooperated multiple times to organize conferences and events together (D40, D41, D95, D98, D174) with Jürgen Elsässer directly receiving money from this institute to finance the events (D98, D95, D112).

Similarly, speculations about direct financial support have also been made concerning the Austrian alternative media outlet *AUF1.tv* (D96, D152), which has close ties to the Kremlin-friendly Austrian right-wing party FPÖ (D36, D48, D50, D92, D95, D138, D152) and belongs to a cluster of other Austrian alternative media outlets, including *Wochenblick, Report24*, and *InfoDirekt* (D48, D92, D95, D138, D152, D153). However, AUF1.tv finances itself mostly via donations (D48, D138, D152). As it offers the opportunity to make anonymous donations via crypto-currencies, the editorial team might not even be aware of who funds its efforts. Moreover, Alina Lipp, the owner and content producer of the outlet *Neues aus Russland* (English: *News from Russia*), serves as a board member in the German branch of the association *Friends of Crimea*, a Kremlin-friendly organization in favor of the annexation of Crimea (D66, D186), while Thomas Röper, who runs the blog *Anti-Spiegel*, served as an “independent” official election observer to the 2022 pseudo-referendum in the Ukrainian oblast of Kherson, which was organized by Russia to formally annex the territory it had conquered earlier in the same year (D116, D184).

Thomas Röper and Alina Lipp are often officially invited to conferences hosted by Kremlin-affiliated organizations or institutions (D66, D72, D73, D113, D116, D176). Prominently, shortly before Russia’s attack on Ukraine in February 2022, the Civic Chamber of the Russian Federation hosted a conference to discuss relations between Russia and Ukraine, where Lipp and Röper were both present, among other pro-Kremlin propagandists, including Darya Dugina, who died in a car bombing in August 2022 and was the daughter of the Russian far-right political philosopher Alexander Dugin (D66, D68, D116). Röper has also been invited to other conferences to speak about alleged war crimes committed by Ukrainian soldiers (D72, D176). Both Lipp and Röper deny a direct affiliation to, as well as financing by Russia or the Kremlin (D66, D67, D68, D103, D166) and are, similar to AUF1.tv, funded by donations whose origins remain largely unknown (D67, D68, D70, D161, D166).

Lastly, meetings between representatives of alternative outlets and representatives of Russia’s ruling elites can be observed. Alina Lipp, for instance, has met with the Director of the Information and Press Department of the Ministry of Foreign Affairs of the Russian Federation, Maria Zakharova, multiple times (D66, D67, D68, D103, D166).

#### Media Connections

The overarching category of media connections entails media or content partnerships of alternative media outlets with Kremlin-affiliated media or organizations as well as frequent unilateral content sharing. First, media or content partnerships include using each other as interview partners, (guest) authors, or experts, frequently sharing the same content or cooperating on content, and frequently referencing each other in articles or posts. Second, unilateral content sharing refers to a one-sided relationship, for example, frequently sharing articles from Kremlin-sponsored outlets, forwarding their posts on Telegram, or advertising for them without receiving anything in return.

Multiple persons and/or outlets are involved in media or content partnerships, either with Kremlin-affiliated media outlets or organizations. For example, Jürgen Elsässer (*Compact*; D40, D173), Alina Lipp (*News from Russia*; D66, D68, D70, D103, D104, D128, D160, D161, D166), Thomas Röper (*Anti-Spiegel*; D103, D116), Michael Mross (*MMnews*; D85, D87), and Ken Jebsen (previously *KenFM*, now *Apolut*; D85, D89, D104, D173) have all been invited to speak as experts on Russian state media or on RT German on various occasions. In return, they frequently refer to Russian state media or RT German in their articles, using them as sources. In 2014, Compact cooperated with an RT German employee for a special magazine edition with a focus on Putin (D28, D187, D188). In addition, the outlet *NachDenkSeiten* shares multiple authors with RT German (D101, D129). After RT German was blocked across the European Union following Russia’s attack on Ukraine, *NachDenkSeiten* provided explanations on how to circumvent the block by accessing RT’s page through a virtual private network (VPN) connection (D54, D189).

Röper and Lipp have served as so-called war correspondents since Russia’s invasion of Ukraine. Accompanied by the Russian military, they travel through Ukrainian territories occupied by Russia and post articles about their travels on their pages and platforms, sometimes even attending exclusive filming and photographing events (D37, D66, D67, D68, D70, D128, D140, D160, D166, D176). Alina Lipp stated that she sells some of the material to Russian state media outlets (D66, D70, D128), indicating a fairly direct line of financing between her and Kremlin-affiliated entities. According to Röper, these trips through temporarily occupied territories are, among others, organized by the Russian Ministry of Defense (D37), outlining the support of the Russian government for Röper’s and Lipp’s content production.

Another form of media partnership exists between *eingeSCHENKt.TV* and the “friendship association” *Druschba-Global*, a (likely) Kremlin-affiliated organization. *EingeSCHENKt.TV* serves as an official media partner and endorser of *Druschba-Global* (D93, D161). *Druschba-Global* organized so-called *friendship trips* from Germany to Russia or the temporarily occupied territories of Ukraine, as well as other events, such as presentations and regional get-togethers (D161, D191). The association maintains connections to the biker club *Night Wolves*, which was involved in Russia’s annexation of Crimea in 2014 (Kleiner et al. 2023; D26, D117, D118, D119, D161). The outlet eingeSCHENKt.TV accompanied some of the friendship trips with a camera team and posted the videos on their website and YouTube channel. This content was also shared by *Klagemauer.TV*, a Swiss alternative media outlet (D93, D111, D161). It is not entirely clear who finances *Druschba-Global*; however, because of their trips and events and their connections to established pro-Kremlin entities, an affiliation between Russia’s elites and Druschba-Global can be assumed.

Concerning unilateral media connections, for example, through frequent one-sided content sharing or advertising, the aforementioned outlet *Klagemauer.TV* advertises for RT German and frequently shares content by Lipp and Röper (D86, D88), while *NachDenkSeiten* has been known to share RT German’s content on its platform (D54, D129). In a similar vein, Bodo Schiffmann, from the channel *Alles Ausser Mainstream* (English: *Everything except mainstream*), hosted RT German’s live stream and extensively shared its content on his Telegram channel after the outlet’s ban. Schiffmann also gave extensive instructions on how to access RT German with a VPN to circumvent its ban (D56, D75, D76, D104, D108, D154, D162, D167).

#### Personal Connections

Lastly, personal connections could be observed between single entities belonging to specific alternative media outlets and Russia’s ruling elites. In contrast to organizational connections, these personal connections refer to unofficial cooperation or activities not specifically related to the outlet. However, the following examples involve people who are highly influential in the selected outlets, which makes personal connections a valuable link for Kremlin-affiliated entities. These personal connections include active or passive event participation, as well as travels to Russia or occupied territories unrelated to the media outlets.

A person with major personal connections to Russia’s elites is Ken Jebsen (*Apolut*). In the past, he personally participated in conferences or events unrelated to his role as content producer; for example, in 2018, he participated in a business event hosted by a Kremlin-affiliated organization (D80, D98, D167), where he apparently advertised housing properties in Crimea. Apart from that, Jebsen repeatedly traveled to Russia or temporarily occupied Ukrainian territories for unknown purposes (D79, D89, D90, D107, D174). Furthermore, Jebsen maintains personal connections to two influential Kremlin-affiliated personalities: Ivan Rodionov (D89, D107), former chief editor of RT German, and Alexander Malkevich (D89, D107), who had implicit connections to Russia’s infamous *IRA*, served as head of the press committee of Russia’s Civic Chamber in 2018, and “took over audiovisual propaganda in the occupied territories of Ukraine in July 2022.”^8^ Jebsen and Malkevich met in 2019 to plan joint projects—more details are however unknown.

The chief editor of the previously mentioned Austrian alternative media outlet *AUF1.tv*, Stefan Magnet, not only maintains close connections to the Kremlin-friendly Austrian party *FPÖ* but also accompanied FPÖ-members on a trip in 2016 to Moscow, where the FPÖ signed a “cooperation pact” with Putin’s party *United Russia* (D48, D95, D152). His specific role in this visit is unknown; however, he met multiple high-ranking Russian figures during the event. Apart from this, Magnet calls himself an “avowed Putin sympathizer” (D95).

### Extent of Connections Between Alternative Media Outlets and Russia’s Ruling Elites

The second part of the research questions asks to what extent the German-language alternative media landscape is connected to Russia’s ruling elites. As can be seen from the previous section, some alternative media outlets maintain connections across multiple dimensions, while other outlets are in no way related to Russia’s ruling elites and are—in some cases—fairly outspoken against Russia’s attack on Ukraine. Table 2 summarizes the occurrence of the three types of connections across the twenty outlets analyzed.

**Table 2.** Overview across analyzed alternative media outlets (*n* = 20) and the three dimensions of connection.

Outlet name	Organizational	Media	Personal
Tichys Einblick			
Reitschuster			
Achse des Guten			
Epoch Times			
PI-News			
Journalistenwatch			
Anti-Spiegel	X	X	
Report24			
NachDenkSeiten		X	
Wochenblick			
Junge Freiheit			
MMnews		X	
DWN			
Apolut/KenFM		X	X
AUF1.tv			X
Compact	X	X	
Kla.tv		X	
Neues aus Russland	X	X	
eingeSCHENKt.TV		X	
Alles Ausser Mainstream		X	

*Note.* A table containing the source IDs for each claim can be found in the Supplemental Table A1.

As Table 2 shows, the majority of connections are located at the media level, referring to media partnerships and content sharing. On the one hand, out of the twenty analyzed outlets, ten did not reveal any connections to Kremlin-affiliated entities—some of them even strongly opposed the Kremlin. Particularly, the chief editors of the outlets *Reitschuster, Junge Freiheit, Achse des Guten*, and *Tichys Einblick* decidedly distanced themselves from Russia’s ruling elites, especially regarding Russia’s full-scale invasion of Ukraine (D7, D8, D13, D21, D108, D120, D124, D183).

Furthermore, the right-wing outlets *PI News* and *Journalistenwatch* take a self-proclaimed pro-American position (D60, D123, D132, D142, D144), even though their understandings of pro-Americanism certainly deserve critical scrutiny. Journalistenwatch, for instance, receives funding from a US-based anti-Islamic think tank, the *Middle East Forum* (D38, D60, D141, D142, D151). These six outlets are some of the most visited prolific German-language alternative media outlets (see Table 1). On the other hand, ten out of the twenty analyzed German-language alternative media outlets are in one or multiple ways connected to Russia’s ruling elites, with some outlets, such as *Compact, Apolut, Anti-Spiegel*, and *News from Russia*, revealing a fairly high connectedness across multiple dimensions.

## Discussion

The goal of this case study was to scrutinize the connections between leading German-language alternative media outlets and Russia’s ruling elite. During the course of the analysis, we identified three kinds of connections: organizational, media, and personal connections. As the findings demonstrate, half of the twenty most popular outlets analyzed maintain at least one kind of connection to Russia’s ruling elites, with the extent ranging from implicit to explicit, providing first insights into the Kremlin’s involvement with media actors abroad.

These findings further emphasize that the Kremlin’s efforts—or more abstractly, efforts by foreign actors—play an important role in the analysis of alternative media landscapes. This role has thus far been largely disregarded. While some studies differentiate between Russia-affiliated and other alternative media outlets, extant research puts a strong emphasis on openly connected outlets, such as RT and Sputnik (Bachl 2018; Frischlich et al. 2022; Ihlebæk et al. 2022; Klawier et al. 2022; Müller and Freudenthaler 2022; Schwarzenegger 2021; Schweiger 2017). However, the Kremlin evidently seeks to influence other alternative media outlets, albeit through other, more opaque means. For future research on alternative media, we suggest broadening the classification of Kremlin-affiliated outlets and using the findings of this case study to more thoroughly consider the various types of connections between alternative media outlets and Russia’s ruling elites. This call is relevant not only for future research on alternative media content but also on organizational structures and audience perception.

Similarly, the extant literature on Russia’s active measures focuses strongly on foreign communication actors, such as RT, Sputnik, or the IRA. Nonetheless, as Abrams (2016) emphasized, Russia’s sphere of influence encompasses much more than these entities, which is confirmed by the findings of this study. As it stands, alternative media need to be considered a potential asset of the Kremlin’s active measures to exert influence abroad, ranging from overt to covert activities. Maintaining such connections might be particularly enticing for the Kremlin since it utilizes already established or seemingly unconnected actors in a foreign media landscape, making the pro-Kremlin narratives appear more innate and organic. In this regard, Kremlin-affiliated alternative media can be understood as agents of influence (DeBenedictis 2022; Shultz and Godson 1984), trying to covertly sway the opinion climate in the alternative media landscape to Russia’s advantage.

This brings into focus multiple important issues concerning the alternative media landscapes of democracies in Western Europe and across the globe. In Germany, for example, the intransparency regarding the economic and financial support of alternative media outlets poses an immense issue, as it often remains uncertain whether these outlets are actually financially supported by the Kremlin for spreading pro-Russian narratives or are merely doing so due to ideological similarities, emphasizing the need for further regulations on ownership and financing transparency. This also ties in with another issue, namely the question of whether support of the Kremlin is witting and unwitting. In the case of several important figures of the German-language alternative media landscape maintaining close relationships with Russia’s ruling elites, such as Jürgen Elsässer or Ken Jebsen, it appears to be their own deliberate choice to cooperate with the Kremlin. By contrast, other actors and outlets, especially those involved in one-sided content sharing, might not necessarily pursue intentions of willfully exerting influence but rather sympathize with certain pro-Russian narratives. This might be based on shared ideology; for example, the Kremlin frequently relies on right-leaning narratives (such as anti-immigration or anti-LGBTQ narratives; see, e.g., Edenborg 2020; Wagnsson and Barzanje 2021), which are also prominently used by right-wing outlets (e.g., Mayerhöffer 2021; Müller and Freudenthaler 2022; Vowles and Hultman 2021). Additionally, opportunism—for example, concerning financial support or the pursuit of a larger audience—might be another driving factor in the (witting) affiliation with the Kremlin. Lastly, due to alternative media’s role as a “self-perceived corrective” (Holt et al. 2019: 862) of the mainstream opinion, they might spread pro-Kremlin content to “balance out” the public discourse.

While the findings of this study focus only on German-language alternative media outlets, similar types of connections and interaction patterns can arguably be expected in other Western countries targeted by Russia’s foreign influence efforts, as both the utilization of foreign communication outlets and pro-Kremlin influencers can be observed across various European countries.^5,6^ Moreover, the question remains whether these findings also apply to communication tactics by other foreign actors, particularly by other authoritarian and repressive regimes. As a report by the *Australian Strategic Policy Institute* indicates, the Chinese government employs similar tactics of covert propagandistic communication (Ryan et al. 2022). By employing so-called *frontier influencers*, the Chinese government spreads regime-friendly narratives from seemingly authentic accounts that are closely linked to government agencies. Thus, the utilization of digital media actors by foreign actors seems to be on the rise across various countries, making the detection of such entities even more imperative.

This study is not without its limitations, which, however, open up promising paths for future research. First, for some outlets, it proved difficult to find detailed information, particularly for niche outlets, such as *MMnews* or *DWN*. That said, the document analysis of this study relied (mostly) on investigative reports and publicly available open-source intelligence. Future research could supplement this knowledge by conducting oral interviews with experts, investigative journalists, or (former) employees of these outlets. Second, in this analysis, we did not seek to assess the strength of the connections or whether the connections were sought by the Kremlin or by the alternative media outlet. We intentionally refrained from doing so due to the scarcity of information on some outlets and the high intransparency and ambiguity that remained regarding specific aspects such as financing or personnel. For example, Alina Lipp openly states being an official “war correspondent” and sells her content to Russian state-sponsored media but, at the same time, denies any affiliation with the Kremlin. Similar concerns with regard to the lack of knowledge and transparency have also been raised by other researchers analyzing covert actions. For instance, as Kux (1985) stated in his work on active measures, “it is frequently difficult to distinguish between legal ‘white’ or ‘gray’ propaganda activities and illegal, clandestine ‘black’ operations” (26). Thus, we refrained from developing a more nuanced assessment of the strength of connections or their directedness, as doing so would inevitably have involved a high degree of freedom in regard to the categorization of the material. That said, grounded in deeper, interview-based analysis, future research may aim to develop more nuanced claims that factor in the intensity, directedness, and motivations of interactions between Kremlin-affiliated actors and alternative media outlets.

Third, in this article, we did not scrutinize the content published by the media outlets. Future research drawing on content analysis can provide nuanced insights into the degree to which the connections to the Kremlin identified in this study affect the coverage of specific outlets. Fourth, this study focused solely on theorizing the Kremlin’s efforts to connect with foreign alternative media outlets. Future research could expand this lens by comparing the types of connections scrutinized in this study to how (Russia’s) authoritarian elites seek to influence (1) foreign legacy media in Western democratic states and (2) domestic media in their authoritarian contexts. In developing these comparisons, they can draw on the literature on media capture (Dragomir 2018; Yanatma 2021), which has scrutinized a range of strategies by which autocrats seek to “capture” their domestic media landscape, including financial incentives, regulation, legislation, physical attacks, and threats against journalists.

To conclude, even though the alternative media landscape changes quickly, and user metrics, as well as relevant platforms, might change in a short time span, this study generated novel and generalizable insights into how, and the degree to which, a Western alternative media landscape is penetrated by connections to Kremlin-affiliated entities. It thus highlights the need for more nuanced approaches to the classification of the Kremlin’s connections in research on alternative media and active measures. Furthermore, the heavy permeation of the Kremlin’s influence on alternative media landscapes should be considered by policymakers concerning regulations and measurements to maintain independent media systems as well as democratic governance.

## Supplemental Material

sj-docx-1-hij-10.1177_19401612241230284 – Supplemental material for Conduits of the Kremlin’s Informational Influence Abroad? How German-Language Alternative Media Outlets Are Connected to Russia’s Ruling Elites Supplemental material, sj-docx-1-hij-10.1177_19401612241230284 for Conduits of the Kremlin’s Informational Influence Abroad? How German-Language Alternative Media Outlets Are Connected to Russia’s Ruling Elites by Arista Beseler and Florian Toepfl in The International Journal of Press/Politics

## Notes

1. Anton Livshits, “Krieg gegen die Ukraine: Wieso „Querdenken“ zu Putin hält. [War against Ukraine: Why “Lateral Thinkers” Stay on Putin’s Side.],” *Belltower.News*. https://www.belltower.news/krieg-gegen-die-ukraine-wieso-querdenken-zu-putin-haelt-129531/

2. Julia Smirnova, and Francesca Arcostanzo. 2022. “German-Language Disinformation about the Russian Invasion of Ukraine on Facebook.” *Digital Dispatches*. https://www.isdglobal.org/digital_dispatches/german-language-disinformation-about-the-russian-invasion-of-ukraine-on-facebook/

3. Sara Bundtzen, and Mauritius Dorn. 2022. “Banning RT and Sputnik Across Europe: What Does It Hold for the Future of Platform Regulation?” *Digital Dispatches*. https://www.isdglobal.org/digital_dispatches/banning-rt-and-sputnik-across-europe-what-does-it-hold-for-the-future-of-platform-regulation/

4. Foo Yun Chee. 2022. “EU Bans RT, Sputnik over Ukraine Disinformation.” *Reuters*. https://www.reuters.com/world/europe/eu-bans-rt-sputnik-banned-over-ukraine-disinformation-2022-03-02/

5. Isolde Ruhdorfer. 2022. “Propaganda auf Social Media: Die Putinfluencer. [Propaganda on Social Media: The Putinfluencers.]” *Die Zeit*. https://www.zeit.de/campus/2022-06/propaganda-social-media-influencer-russland?page=4&utm_referrer=https%3A%2F%2Fwww.google.com%2F

6. Brandy Zadrozny. 2022. “Russian Propaganda Efforts Aided by Pro-Kremlin Content Creators, Research Finds.” *NBC News*. https://www.nbcnews.com/tech/tech-news/russian-propaganda-efforts-aided-kremlin-content-creators-research-fin-rcna32343

7. The IDs refer to the documents in which the discussed information is provided. The documents can be found in the appended Supplemental Information file.

8. Reporters without Borders. 2023. “The Malkevich Propaganda Machine” *RSF*. https://rsf.org/en/investigation-mysterious-alexander-malkevich-kremlin-propagandist-ukraine
