# Supplementary material for: Conduits of the Kremlin’s Informational Influence Abroad? How German-Language Alternative Media Outlets Are Connected to Russia’s Ruling Elites
Source: Int J Press Polit. 2024 Feb 20;30(3):659–78. doi: 10.1177/19401612241230284 (PMC12145180; doi:10.1177/19401612241230284)
Supplement: sj-docx-1-hij-10.1177_19401612241230284 – Supplemental material for Conduits of the Kremlin’s Informational Influence Abroad? How German-Language Alternative Media Outlets Are Connected to Russia’s Ruling Elites [file sj-docx-1-hij-10.1177_19401612241230284.docx]

**Appendix: Supplementary Information**

**Table A1.** Overview across Analyzed Alternative Media Outlets (n = 20) and the Three Types of Connection with Document IDs

|  | Organizational | Media | Personal |
| --- | --- | --- | --- |
| Tichys Einblick |  |  |  |
| Reitschuster |  |  |  |
| Achse des Guten |  |  |  |
| Epoch Times |  |  |  |
| PI-News |  |  |  |
| Journalistenwatch |  |  |  |
| Anti-Spiegel | D66, D72, D113, D116, D176, D184 | D37, D68, D70, D103, D116, D140, D176 |  |
| Report24 |  |  |  |
| NachDenkSeiten |  | D54, D101, D128, D129, D189 |  |
| Wochenblick |  |  |  |
| Junge Freiheit |  |  |  |
| MMnews |  | D85, D87 |  |
| DWN |  |  |  |
| Apolut / KenFM |  | D85, D89, D104, D107, D161, D173 | D79, D80, D89, D90, D98, D107, D167, D174 |
| AUF1.tv |  |  | D48, D95, D152 |
| Compact | D22, D39, D40, D41, D95, D98, D112, D174 | D28, D40, D173, D187, D188 |  |
| Kla.tv |  | D86, D88, D111 |  |
| Neues aus Russland | D66, D68, D103, D116, D161, D166, D176, D186 | D66, D67, D68, D70, D103, D104, D128, D140, D161, D166, D176 |  |
| eingeSCHENKt.TV |  | D93, D161 |  |
| Alles Ausser Mainstream |  | D56, D75, D76, D108, D154, D162, D167 |  |

**Information about the Analyzed Outlets**

**Tichys Einblick** (https://www.tichyseinblick.de/) is one of the largest German-language alternative media outlets and is located in Germany. The chief editor, Roland Tichy, used to work as a journalist for the German economy magazine *Wirtschaftswoche*. Tichys Einblick covers multiple positions on the political spectrum, many articles are however right-leaning.

**Reitschuster** (https://reitschuster.de/) is run by Boris Reitschuster who used to lead the international office for the German magazine Focus in Moscow. He is very critical of Putin and his regime. The outlet is located in Germany and can be described as right-leaning and establishment-critical.

**Achse des Guten** (https://www.achgut.com/) can be considered a blog and its content is mostly located on the right end of the political spectrum. It’s located in Germany but also gives a plethora of guest authors the possibility to publish on the website.

**Epoch Times** (https://www.epochtimes.de/), in this case the German-language version, is a multi-lingual online newspaper with Chinese-American origin and connections to the Falun Gong movement. Politically it is located on the right side of the spectrum.

The blog **PI-News** (https://www.pi-news.net/) publishes far-right content, with a particular focus on anti-Islamic content. Due to its extremism, PI-News is subject to observation by the German domestic intelligence service. The current owner of the platform is unknown.

**Journalistenwatch** (https://journalistenwatch.com/) publishes content in German language and is located in Hungary. The content ranges from right to far-right. The website receives funding from an Islamophobic think tank located in the US.

**Anti-Spiegel** (https://www.anti-spiegel.ru/) is run by Thomas Röper, a German living in Saint Petersburg, Russia. On this website, Röper publishes pro-Russian propagandistic content, including disinformation, and conspiracy narratives. Since July 2023, Röper openly cooperates with RT German for the format Anti-Spiegel TV.

**Report24** (https://report24.news/) belongs to the alternative media cluster of Austrian outlets, sharing employees with *Wochenblick* as well as *AUF1.tv*. The content contains conspiracy narratives and misinformation. It is politically situated on the right.

**NachDenkSeiten** (https://www.nachdenkseiten.de/) is a blog located in Germany. The owner, Albrecht Müller, used to be a member of the German political party *SPD* (Social-democratic party). While the website publishes content from various political positions, the content is anti-establishment and also entails conspiracy narratives.

Based in Austria, **Wochenblick** (https://www.wochenblick.at/) also belonged to the Austrian alternative media cluster, sharing, among others, conspiracy narratives and disinformation. It maintained close ties to the right-wing Austrian political party *FPÖ*. In December 2022, *Wochenblick* was closed due to financial reasons.

The right-wing newspaper **Junge Freiheit** (https://jungefreiheit.de/) is located in Germany and publishes print versions as well as online articles. The articles range from conservative to far-right, covering a spectrum of right-wing perspectives.

The outlet **MMnews** (https://www.mmnews.de/) which is run by a former German journalist, Michael Mross, who previously worked for various German media outlets, puts a strong focus on news about the economy. Additionally, some content features conspiracy narratives and anti-Semitic narratives.

Similar to MMnews, **Deutsche Wirtschafts Nachrichten** (DWN, https://deutsche-wirtschafts-nachrichten.de/) has a thematic focus on the economy. It is located in Germany. While the website used to feature dubious content, its articles have become more balanced in recent years.

**Apolut** (https://apolut.net/) is the new outlet established by Ken Jebsen, who became famous in the alternative media landscape with his outlet **KenFM**. Ken Jebsen used to work as a radio host in Berlin, Germany, but was fired due to allegations of anti-Semitism. As a reaction, he founded *KenFM*. In 2021, Ken Jebsen closed KenFM after legal disputes with a German media oversight institution due to KenFM’s violations of the press code. *Apolut* now hosts various formats with guest authors with many articles or videos containing conspiracy narratives and disinformation.

**AUF1.tv** (https://auf1.tv/) is another outlet belonging to the cluster of Austrian alternative media websites. Even though it is located in Austria, many of the articles focus on Germany or German politics. The content can be classified as far-right and also features conspiracy narratives, disinformation, anti-Semitism and pro-Russian propaganda. The outlet maintains close ties to the Austrian right-wing party *FPÖ*.

The outlet **Compact** (https://www.compact-online.de/) is well-established in the German far-right scene and has been subject of observation by the German domestic intelligence service since 2021. The outlet features content which contains conspiracy narratives, anti-Semitism, Islamophobia, anti-LGBTQ sentiment, and pro-Russian propaganda.

**Kla.tv** (short version of **Klagemauer.tv**, https://www.kla.tv/) is located in Switzerland. The outlet is connected to the religious sect *Organische Christus Generation* (OCG, Organic Christ Generation) and features far-right content as well as right esoterism, conspiracy narratives, and anti-Semitism.

**Neues aus Russland** (https://neuesausrussland.com/, **News from Russia**), is a blog run by Alina Lipp, a German citizen born in Hamburg with a Russian migration background who emigrated to Russia or temporarily occupied territories in Ukraine in 2021. Mostly famous for her Telegram channel, she posts content about Russia’s full-scale war against Ukraine with a distinct pro-Russian perspective. She uses disinformation and conspiracy narratives in line with pro-Russian propaganda and sometimes posts content from battle zones.

The right-wing esoteric outlet **eingeSCHENKt.tv** (https://eingeschenkt.tv/) cooperates with *AUF1.tv* for certain video formats. Similar to AUF1.tv, the outlet eingeSCHENKt.tv features content with conspiracy narratives. Also, they accompanied trips to Russia or temporarily occupied territories in Ukraine in order to shoot video footage.

Lastly, the Telegram channel **Alles Ausser Mainstream** (https://t.me/s/AllesAusserMainstream) is run by a German physician, Bodo Schiffmann. He became famous during the COVID-19 pandemic when he posted conspiracy narratives surrounding the disease and the vaccine. He also features QAnon narratives on his channel as well as statements that trivialized the holocaust.

**Document Collection for Case Study** (sorted by ID)

*Note.* The MAXQDA file used for coding is available upon request. All texts were archived as PDF file or MAXQDA Web Collector file and are also available upon request.

D1: Schmidt, N. (17 December 2017). Die Amerika-Connection der Neuen Rechten. *ZEIT ONLINE.* <https://www.zeit.de/kultur/2017-12/journalistenwatch-neue-rechte-finanzierung>

D2: Weiland, S. (24 February 2022). Schock mit Ansage. *SPIEGEL Plus.* <https://advance.lexis.com/api/document?collection=news&id=urn:contentItem:64VX-NXN1-JBR8-40W4-00000-00&context=1516831>

D3: Weiland, S. (20 March 2019). Wer holte Billy Six aus Venezuela? *DER SPIEGEL.* <https://www.spiegel.de/politik/deutschland/venezuela-wer-sorgte-fuer-die-freilassung-von-billy-six-a-1258622.html>

D4: Fiedler, M., & von Salzen, C. (9 April 2019). Russlands Spiel mit den Rechten. Dokumente zeigen, wie Moskau auf die AfD; Einfluss nehmen wollte. Worum geht es, und welche Strategie verfolgt der Kreml? *Der Tagesspiegel*. <https://advance.lexis.com/api/document?collection=news&id=urn:contentItem:5VV6-N6K1-F066-F11C-00000-00&context=1516831>.

D5: Kluge, C. (27 July 2020). Das Eva-Prinzip Wie die einst so beliebte Fernsehmoderatorin Eva Herman in die rechte Szene abgedriftet ist. *Der Tagesspiegel.* <https://advance.lexis.com/api/document?collection=news&id=urn:contentItem:60FG-TDK1-JCR4-R0KV-00000-00&context=1516831>.

D6: Hock, A. (18 January 2019). Vorwürfe; AfD-Mitarbeiter im Bundestag nach Berichten über Anschlag entlassen. *WELT ONLINE*. <https://advance.lexis.com/api/document?collection=news&id=urn:contentItem:5V79-VJ61-F16N-0133-00000-00&context=1516831>.

D7: Broder, H. M. (14 March 2022). Das Verhalten der Nato ist unterlassene Hilfeleistung. *DIE WELT.* <https://advance.lexis.com/api/document?collection=news&id=urn:contentItem:650G-MHW1-JBK9-200Y-00000-00&context=1516831>.

D8: Kamann, M. (1 February 2019). Rede bei der AfD; Broder hat Richtiges gesagt, aber auch Gefährliches befeuert. *Bilanz.de.* <https://advance.lexis.com/api/document?collection=news&id=urn:contentItem:5VB4-JMP1-JBK9-244J-00000-00&context=1516831>.

D9: Broder, H. M. (4 May 2022). Endlich gibt es einen Sündenbock; Die Ukrainer sollen deeskalieren, damit die Russen nicht auf die Idee kommen, Atomwaffen einzusetzen. Und falls sie es doch tun, werden die Ukrainer dafür verantwortlich sein. Eine dialektische Meisterleistung. *DIE WELT.* <https://advance.lexis.com/api/document?collection=news&id=urn:contentItem:65C7-YV71-DY2B-S50R-00000-00&context=1516831>.

D10: Maxeiner, D., & Miersch, M. (2 May 2014). Streiken gegen Putin. *DIE WELT.* <https://advance.lexis.com/api/document?collection=news&id=urn:contentItem:5C3V-CTJ1-JBK9-21KJ-00000-00&context=1516831>.

D11: Bommarius, C. (31 January 2019). An das deutsch-nationale Pöbel-Pack. *Frankfurter Rundschau.* <https://www.fr.de/kultur/deutsch-nationale-poebel-pack-11630319.html>

D12: Broder, H. M. (21 November 2007). Kanzlerstreit: Dem Putin dienen. *SPIEGEL ONLINE.* <https://www.spiegel.de/politik/ausland/kanzlerstreit-dem-putin-dienen-a-518606.html>

D13: Tichy, R. (6 April 2022). Die unerwiderte Liebe des Hufeisens zu Wladimir Putin. *TICHYS EINBLICK.* <https://www.tichyseinblick.de/tichys-einblick/die-unerwiderte-liebe-des-hufeisens-zu-wladimir-putin/>

D14: Ringler, N., Schacht, K., Schnuck, O., & Schöffel, R. (8 December 2016). BR Data: Rechtes Netz. *BR.de.* <https://interaktiv.br.de/rechtes-netz/>

D15: Fleischhauer, J. (21 May 2022). Die Enden des Hufeisens. *FOCUS.* <https://www.wiso-net.de/document/FOCU__5de9da5ee924177e14c7b86c40a107472d56fb44>

D16: Seifert, S. (3 March 2018). Dieser Mann soll schweigen. *taz.die tageszeitung.* <https://www.wiso-net.de/document/TAZ__dfb6b537e77f26bc5f05c20fb7ab62721a291b34>

D17: Greive, M., Hildebrand, J., & Neuerer, D. (24 September 2020). Nach frauenverachtendem Artikel: Roland Tichy gibt Vorsitz der Ludwig-Erhard-Stiftung ab. *Handelsblatt.* <https://www.handelsblatt.com/politik/deutschland/umstrittene-aeusserungen-nach-frauenverachtendem-artikel-roland-tichy-gibt-vorsitz-der-ludwig-erhard-stiftung-ab/26214854.html>

D18: Tichy, R. (31 May 2015). Warum es falsch ist, dass Putin bei G7 ausgesperrt wird. *Bild am Sonntag.* <https://www.wiso-net.de/document/BISO__482612905bc8b3cf39d7cf8ded230f68da04d981>

D19: Huesmann, F. (30 March 2022). Gegen Impfpflicht und für Putin. *Frankfurter Rundschau.* <https://www.wiso-net.de/document/FR__806fc9402d14a4d6f492eab24ac515646da00680>

D20: Götschenberg, M. (10 December 2021). Verfassungsschutz zu “Compact”: “Gesichert extremistisch”. *tagesschau.de.* <https://www.tagesschau.de/inland/innenpolitik/compact-magazin-101.html>

D21: Reveland, C., & Siefert, V. (4 March 2022). Russlands Krieg gegen die Ukraine: “Querdenker” für Putin. *tagesschau.de.* <https://www.tagesschau.de/investigativ/reaktionen-auf-putin-von-querdenkern-und-verschwoerungsideologen-101.html>

D22: Gensing, P., & Stöber, S. (29 April 2016). Pro-russische Netzwerke: Moskautreue Rechte. *tagesschau.de.* <https://www.tagesschau.de/inland/neurechte-russland-101.html>

D23: Reinhard, O. (9 July 2021). Ein “nützlicher Idiot” des Kreml. *Sächsische Zeitung.* <https://www.wiso-net.de/document/SZO__80169711c0b346905cf7e4a4d838632a995ce1a4>

D24: Baumgärtner, M., Müller, A.-K., & Traufetter, G. (28 May 2022). Spiel mit der Angst. *DER SPIEGEL.* <https://www.wiso-net.de/document/SPIE__PMG4SPIEGEL-Heftimport-SP20220528-92685_872887d2-4e64-4c94-82c2-7fc507437070%7CTSPI__PMG4SPIEGEL-Heftimport-SP20220528-92685_872887d2-4e64-4c94-82c2-7fc507437070>

D25: Middelhoff, P., & Musharbash, Y. (5 May 2022). … aber unsere Liebe nicht. *DIE ZEIT.* <https://www.wiso-net.de/document/ZEIT__FDAE4A9E2A4D5893D9FFFE44C60EB2DF%7CZEIA__FDAE4A9E2A4D5893D9FFFE44C60EB2DF>

D26: Joswig, G. (29 March 2022). Wie hältst du’s mit Russland? *taz.die tageszeitung.* <https://www.wiso-net.de/document/TAZ__4bcb74a87ba5d0c3d73b30963de570ba53e2f4f2>

D27: Hoffmeister, H., Müller, A.-K., Röbel, S., & Wiedmann-Schmid, W. (26 March 2022). Putins fünfte Kolonne. *DER SPIEGEL.* <https://www.wiso-net.de/document/SPIE__PMG4SPIEGEL-Heftimport-SP20220326-71247_64cfd8f3-2735-4047-857b-0249940ad8cb%7CTSPI__PMG4SPIEGEL-Heftimport-SP20220326-71247_64cfd8f3-2735-4047-857b-0249940ad8cb>

D28: Baumgärtner, M., Höfner, R., & Müller, A.-K. (27 February 2021). Moskaus Querdenker. *DER SPIEGEL.* <https://www.wiso-net.de/document/SPIE__CODESCO-SP-2021-009-89328%7CTSPI__CODESCO-SP-2021-009-89328>

D29: n.a. (11 August 2017). AfD veranstaltet Kongress zu Russland. *Magdeburger Volksstimme.* <https://www.wiso-net.de/document/MBVS__125430720bae1a6cefd952c1266c535b46cac0d5>

D30: n.a. (n.d.). Russlandkongress der AfD-Fraktion in Magdeburg am 12. August 2017. *AfD-Fraktion im Landtag Sachsen-Anhalt.* <https://www.afdfraktion-lsa.de/termine/russlandkongress-der-afd-fraktion-in-magdeburg-am-12-august-2017/>

D31: Bartz, J., Hano, J., & Stoll, U. (9 April 2019). Der Fall Frohnmaier. Wie russische Strategen einen AfD-Politiker lenken wollten. *ZDF frontal.* <https://www.zdf.de/politik/frontal/der-fall-frohnmaier-100.html>

D32: Katehon. (2 October 2016). Vorwahlen im Donbass: Erfolgreiche Beobachtungsmission. *geopolitica.ru.* <https://www.geopolitika.ru/de/news/vorwahlen-im-donbass-erfolgreiche-beobachtungsmission>

D33: Stöber, S. (12 August 2017). AfD-Russland-Kongress: Experte mit fragwürdiger Vergangenheit. *tagesschau.de.* <https://www.tagesschau.de/faktenfinder/afd-russland-kongress-101.html>

D34: n.a. (30 March 2022). AfD in Bayern: Zum Teil auf Moskaus Linie? *tagesschau.de.* <https://www.tagesschau.de/investigativ/br-recherche/afd-russland-109.html>

D35: n.a. (26 June 2020). Bayerischer Landtag: AfD beschäftigt Russland-Lobbyisten. *tagesschau.de.* <https://www.tagesschau.de/investigativ/afd-landtag-bayern-101.html>

D36: Winter, J. (4 September 2021). Rechtsextremer Corona-Leugner bekam Auftrag von Land Oberösterreich. *profil.* <https://www.profil.at/oesterreich/rechtsextremer-corona-leugner-bekam-auftrag-von-land-oberoesterreich/401724315>

D37: Röper, T. (11 July 2022). Diese Woche fahre ich wieder in den Donbass. *ANTI-SPIEGEL.* <https://www.anti-spiegel.ru/2022/diese-woche-fahre-ich-wieder-in-den-donbass/>

D38: Schmidt, F. (26 March 2019). Wie die neuen Rechten sich vernetzen und woher das Geld kommt. *DER SPIEGEL.* <https://www.spiegel.de/politik/deutschland/endlich-wieder-stolz-sein-wie-die-neuen-rechten-sich-vernetzen-und-woher-das-geld-kommt-a-0b98c5ad-defe-48af-9f4c-4fa35c7bf703>

D39: Bidder, B., & Wittrock, P. (24 November 2014). Das rechte Netz des Kreml. *DER SPIEGEL.* <https://www.spiegel.de/politik/ausland/afd-und-front-national-putin-umwirbt-europas-rechtspopulisten-a-1004746.html>

D40: Korn, T., & Umland, A. (19 July 2014). Jürgen Elsässer, Kremlpropagandist. *ZEIT ONLINE.* <https://www.zeit.de/politik/deutschland/2014-07/juergen-elsaesser-russland-propaganda/>

D41: Langer, A. (23 November 2013). Krude Thesen bei Homophoben-Veranstaltung. *DER SPIEGEL.* <https://www.spiegel.de/panorama/gesellschaft/compact-veranstaltung-krude-thesen-der-homosexuellenhasser-a-935310.html>

D42: Boeselager, M. (27 November 2015). Wie seriös sind die “Deutschen Wirtschafts Nachrichten”? *VICE Deutschland.* <https://www.vice.com/de/article/8gb7dk/wie-serioes-sind-die-deutschen-wirtschafts-nachrichten-283>

D43: Stöcker, C. (4 December 2016). Das Erfolgsproblem der europäischen Rechten. *DER SPIEGEL.* <https://www.wiso-net.de/document/SPON__021ccc5debc4bbef5a99fc9f7f9bbd79d885fa4b>

D44: Kech, F. (7 February 2017). Die halbe Wahrheit - und nicht einmal das - Fake News werden oft von politisch weit rechts stehenden Internetseiten verbreitet / Sie wirken, auch ohne dass sie geglaubt werden. *Badische Zeitung.* <https://www.wiso-net.de/document/BADZ__0233b04bd8ba780ae5f3da1c127158b2a281a837>

D45: Petter, J. & Laufer, D. (11 April 2020). Virale Propaganda: Wie junge Rechtsradikale Buzzfeed kopieren wollen. *DER SPIEGEL.* <https://www.wiso-net.de/document/SPON__d30d002f7e59c86666d0fa168f0171035f67a0f7>

D46: Weisskircher, M. (2020). Neue Wahrheiten von rechts außen? Alternative Nachrichten und der „Rechtspopulismus“ in Deutschland. *Forschungsjournal Soziale Bewegungen*, *33*(2), 474-490. <https://doi.org/10.1515/fjsb-2020-0040>

D47: Braun, K. (23 April 2023). Putins Influencer sitzen auch in Deutschland. *Münchner Merkur.* <https://www.wiso-net.de/document/MUME__acc1dda989833bc7d6509b7833988d94ab0cb7ff>

D48: Horaczek, N. & Tóth, B. (9 March 2022). Das russische Virus. *Falter.* <https://www.wiso-net.de/document/FALT__60b29a7759be1c788bcf15998b029c3a06109191>

D49: Weiser U. & Neuhauser, J. (5 February 2022). Ein Jahr MFG: Was kann die Partei - außer Pandemie-Protest? *Die Presse.* <https://www.wiso-net.de/document/PRE__5b6f431313e48f70824b1f7f008e062748710fe0>

D50: Tóth, B. (20 July 2022). Willkommen im Verschwörungsfunk. *Falter.* <https://www.wiso-net.de/document/FALT__f4da8611312759c9ccfd8de6ac1e86ab46219f43>

D51: Kittel, O. (24 April 2017). Die desinformierten Bürger. *Sächsische Zeitung.* <https://www.wiso-net.de/document/SZO__24f1940aa668e442c1757c64a29d449307b5a019>

D52: Linden, M. (10 October 2015). Die Stimmen des digitalen Untergrunds. *Neue Zürcher Zeitung.* <https://www.wiso-net.de/document/NZZ__9d9fa90e68586eaa5283251f2a9f45c481d7548c>

D53: Kremer, C. (21 May 2022). Der Informationskrieg. *Trierischer Volksfreund.* <https://www.wiso-net.de/document/TV__a1ef583b126597ebcf2f2cbae7428c2f4edc1d90>

D54: Korfmann, M. & Onkelbach, C. (11 April 2022). Putins Propagandamaschine. *Kölnische Rundschau.* <https://www.wiso-net.de/document/KR__MDS-A-19A0C6E4-4220-4067-8D28-55364E13E717-KOELN%7CKRT__MDS-A-19A0C6E4-4220-4067-8D28-55364E13E717-KOELN>

D55: Gentner, P. & Bartsch-Hauschild, T. (25 February 2022). Ein Angriffskrieg auf europäischem Boden. Zum Einmarsch russischer Truppen in die Ukraine und zu "Separatistenführer bitten Putin um Hilfe", FR-Thema von 24.2. *Frankfurter Rundschau.* <https://www.wiso-net.de/document/FR__acebad7811dc9dde29245189282fef205f51e661>

D56: Jakob, C. (12 March 2022). Das reaktionäre “Bauchgefühl”. *taz.die tageszeitung.* <https://www.wiso-net.de/document/TAZ__cc9f9b61336645491848fa9c09e3fba582de64bd>

D57: Geyer, S. (11 December 2014). Seltsame Allianzen. *Mitteldeutsche Zeitung.* <https://www.wiso-net.de/document/MZ__6f0c953dbe609d353991a416cebf9224b20bb2b8>

D58: Kittel, O. (13 April 2021). Welcher Quelle kann man trauen? *Sächsische Zeitung.* <https://www.wiso-net.de/document/SZO__eb88b91381566e092ce96d19ad1df857367c03cb>

D59: Jungkunz, A. (14 April 2022). Putins Informationskrieg. *Nürnberger Zeitung.* <https://www.wiso-net.de/document/NZ__352215caa7246bd742b310f6e12e2661c52a724d>

D60: Schmidt, N. (17 December 2017). Die Amerika-Connection der Neuen Rechten. *ZEIT ONLINE*. <https://www.wiso-net.de/document/ZEIO__9309745b9c5cebaf94adb78102b784e316ee72c0>

D61: Herber, B. (14 September 2017). Eine stetige Quelle der Wut. *DIE ZEIT.* <https://www.wiso-net.de/document/ZEIT__D1EDD50DD2321CEE6FFA6B988FEAAAC2%7CZEIA__D1EDD50DD2321CEE6FFA6B988FEAAAC2>

D62: Fischer, K. (12 March 2016). Kasse machen mit den Rechtspopulisten. *Wirtschaftswoche online.* <https://www.wiso-net.de/document/WWON__33aa08abeafd4b650a0102c761a598dd467ad5cd>

D63: n.a. (28 June 2011). Eine Zeitung, die aus China kommt. Freier Wähler lässt in Frankfurt die People’s Daily erscheinen. *Frankfurter Neue Presse.* <https://www.wiso-net.de/document/FNP__fc46362b830ace108092d141ec7b0eb61b57bfb5>

D64: Meisner, M. (21 March 2022). Virale Desinformation. *taz.de.* <https://taz.de/Fake-Video-ueber-vermeintliche-Toetung/!5840206/>

D65: Drewello, M. (9 June 2022). Putin-Propaganda: Wie deutsche und internationale Influencer die Blockade russischer Medien unterlaufen. *stern.* <https://www.stern.de/politik/ausland/ukraine-krieg--wie-blogger-die-blockade-russischer-medien-unterlaufen-31935048.html>

D66: Wienand, L. (19 April 2022). Putins deutsche Infokriegerin. *t-online.* <https://www.t-online.de/nachrichten/ausland/id_91759336/alina-lipp-auf-telegramm-einst-bei-den-gruenen-jetzt-putins-infokriegerin-.html>

D67: Ruhdorfer, I. (8 June 2022). Die Putinfluencer. *ZEIT ONLINE.* <https://www.wiso-net.de/document/ZEIO__060ec93753971c9ae8425ea4de37544aa59ddfc7>

D68: Reinhard, O. (21 June 2022). Putins deutsche Info-Kriegerin. *Sächsische Zeitung.* <https://www.wiso-net.de/document/SZO__d8ee96a51787a8a2b0749024acef3929750c6245>

D69: Hahn, S. (18 April 2022). Ehemalige Grüne macht Putin-Propaganda in Deutschland. *Kölner Stadt-Anzeiger.* <https://www.ksta.de/politik/ukraine-ex-gruene-alina-lipp-mit-propaganda-fuer-wladimir-putin-266583>

D70: Maus, A., Meyer, L., & Schreiber, A. (21 April 2022). Putins Influencer: Russische Propaganda auf dem Vormarsch. *Das Erste.* <https://www1.wdr.de/daserste/monitor/sendungen/putins-influencer-100.html>

D71: n.a. (27 May 2003). ОТСТАВКИ И НАЗНАЧЕНИЯ. *Ведомости.* <https://www.vedomosti.ru/newspaper/articles/2003/05/27/otstavki-i-naznacheniya>

D72: Буккер, И. (20 June 2022). Хакеры добыли доказательства существования биолабораторий на Украине. *pravda.ru.* <https://www.pravda.ru/world/1720375-ukraina_biovoina/>

D73: Dornblüth, G. (19 April 2022). Alina Lipp und der Krieg: Deutsche „Friedensjournalistin“ als Sprachrohr Putins. *Deutschlandfunk.* <https://www.deutschlandfunk.de/alina-lipp-und-der-krieg-deutsche-friedensjournalistin-als-sprachrohr-putins-dlf-414308a5-100.html>

D74: Kagermeier, E. (25 March 2022). Warum Querdenker nun prorussische Propaganda verbreiten. *Bayerischer Rundfunk.* <https://www.br.de/nachrichten/deutschland-welt/warum-viele-querdenker-nun-prorussische-propaganda-verbreiten,T10vAvf>

D75: Eder, S. (5 March 2022). Warum russische Propaganda bei Corona-Leugnern in Deutschland ankommt. *Frankfurter Allgemeine Zeitung.* <https://www.faz-biblionet.de/fazPortal/saveSingleDoc?explicitId=FAZN__20220305_7854543>

D76: Behme, P. (8 March 2022). Pro-russische Propaganda: Die „Querdenken“-Szene findet neue Narrative. *Deutschlandfunk.* <https://www.deutschlandfunk.de/die-querdenken-szene-findet-neue-narrative-100.html>

D77: Kid, M. (8 March 2022). Nicht nur auf Telegram: Wenn Corona-Skeptiker zu Putin-Verstehern werden – Interview mit Ingrid Brodnig. *Deutschlandfunk.* <https://www.deutschlandfunk.de/wenn-corona-skeptiker-zu-putin-verstehern-werden-interview-mit-ingrid-brodnig-dlf-903950cf-100.html>

D78: Manakas, M. (22 March 2022). Russlands Macht über das deutschsprachige Verschwörungsmilieu. *Der Standard.* <https://www.derstandard.de/story/2000134291184/russlands-macht-ueber-das-deutschsprachige-verschwoerungsmilieu>

D79: Pfahler, L. (8 March 2022). Ein Linker auf Putins Mission. *Die Welt.* <https://www.welt.de/politik/deutschland/article237313489/Prorussische-Querfront-Ein-Linker-auf-Putins-Mission.html>

D80: Kubeth, L. (23 March 2022). PR für Putin: Ken Jebsen bei Corona-Protest in Bautzen. *Sächsische Zeitung.* <https://www.wiso-net.de/document/SZO__a7dc8b62be6528daf4e7e276f1ff63584eb472a6>

D81: n.a. (23 November 2019). 17. AZK-Konferenz. *Ivo Sasek.* <https://www.ivo-sasek.ch/azk-17/>

D82: kla.tv. (19 March 2022). Deutschland im Krieg gegen Russland (von Ivo Sasek). *kla.tv.* <https://www.kla.tv/UkraineKonflikt/21969&autoplay=true>

D83: kla.tv. (3 August 2014). AZK 10: Der Krieg gegen Russland. *kla.tv.* <https://www.kla.tv/UkraineKonflikt/3814&autoplay=true>

D84: n.a. (30 November 2016). Verschwörungs-Sekte: Wir erlösen uns von dem Bösen. *Bayerischer Rundfunk.* <https://www.br.de/nachricht/verschwoerung-sekte-klagemauer-tv-100.html>

D85: Mohr, R. (28 December 2014). Putins deutsche Stimme. *Die Welt.* <https://www.welt.de/print/wams/politik/article135787919/Putins-deutsche-Stimme.html>

D86: kla.tv. (25 February 2022). Zeugenbericht aus Donezk (Ukraine): „Seit 8 Jahren ist hier Krieg!“ (24.2.22). *kla.tv.* <https://www.kla.tv/UkraineKonflikt/21734&autoplay=true>

D87: n.a. (22 December 2014). Der Fehlende Part: Rezession oder kleine Krise. Wo steht Russland? [E 29]. *RT DE.* <https://pressefreiheit.rtde.live/8561/der-fehlende-teil/8561>

D88: kla.tv. (17 June 2014). RT - Russia Today. *kla.tv.* <https://www.kla.tv/3390>

D89: Giese, J. (n.d.). Cui Bono: WTF happened to Ken Jebsen? - Episode 5: Der nützliche Idiot. *DocPlayer.* <https://docplayer.org/222667578-Cui-bono-wtf-happened-to-ken-jebsen.html>

D90: Holland-Letz, M. (31 March 2022). Krise der Demokratie: Wem nutzt Ken Jebsen? *Gewerkschaft Erziehung und Wissenschaft (GEW).* <https://www.gew.de/aktuelles/detailseite/wem-nutzt-ken-jebsen>

D91: Marinov, V., & Bau, M. (14 April 2022). Sie wurde Sprachrohr russischer Propaganda. *M - Menschen Machen Medien.* <https://mmm.verdi.de/internationales/sie-wurde-sprachrohr-russischer-propaganda-80779>

D92: Lenze, D. (29 July 2022). Feindliche Einflussnahme: Die AfD setzt auf neue rechte Medien und Kooperationen. *nd.Aktuell.* <https://www.nd-aktuell.de/artikel/1165710.afd-medien-feindliche-einflussnahme.html>

D93: n.a. (n.d.). 2017 Tour Kaukasus. *Druschba-Global.* <https://druschba-global.org/kaukasus/>

D94: n.a. (n.d.). eingeschenkt.tv AUF1. *AUF1.tv.* <https://auf1.tv/eingeschenkt-tv-auf1>

D95: Sulzbacher, M. (20 July 2022). "Auf 1", "Report 24" und "Wochenblick": Russische Propaganda aus Oberösterreich. *Der Standard.* <https://www.derstandard.at/story/2000137564718/auf-1-report-24-und-wochenblick-russische-propaganda-aus-oberoesterreich>

D96: Kahlweit, C. (21 July 2022). Österreich: Rechtes Portal will nach Deutschland expandieren. *Süddeutsche Zeitung.* <https://www.sueddeutsche.de/medien/auf1-deutschlandplaene-1.5625578>

D97: n.a. (19 March 2019). Russen halfen Billy Six? *Frankfurter Allgemeine Zeitung.* <https://www.faz-biblionet.de/faz-portal/document?uid=FAZ__FD2201903195664403_1>.

D98: Steinke, R. (28 May 2022). Rechtsextremismus: Die Moskau-Connection. *Süddeutsche Zeitung.* <https://www.sueddeutsche.de/politik/russland-rechtsextremismus-unterstuetzung-1.5592490?reduced=true>

D99: Hans, J. (2 September 2015). Russische Propaganda in Deutschland: Liniengrüße aus Moskau. *Süddeutsche Zeitung.* <https://www.sueddeutsche.de/medien/russische-propaganda-in-deutschland-liniengruesse-aus-moskau-1.2630243>

D100: Hanfeld, M. (5 March 2022). Die Realität sieht ganz anders aus. *Frankfurter Allgemeine Zeitung.* <https://www.faz-biblionet.de/faz-portal/document?uid=FAZ__FD22022030550001384704551>.

D101: Hanfeld, M. (3 March 2022). Waffen im Informationskrieg. *Frankfurter Allgemeine Zeitung.* <https://www.faz-biblionet.de/faz-portal/document?uid=FAZ__FD22022030350001374037148>.

D102: Amler, F. (12 September 2014). Friedensbruch. *Süddeutsche Zeitung (Landkreis Wolfratshausen)*, R7.

D103: Kampf, L., Koopmann, C., & Weinmann, L. (8 June 2022). Krieg in der Ukraine: Putins Stimmen im Westen. *Süddeutsche Zeitung.* <https://www.sueddeutsche.de/politik/russland-ukraine-propaganda-influencer-1.5599762>

D104: Kirschbaum, L. (18 March 2022). Putins Plattformprediger. *Frankfurter Allgemeine Zeitung.* <https://www.faz-biblionet.de/faz-portal/document?uid=FAZ__FD12022031850001434234322>.

D105: Burger, R. (22 March 2022). Fake News über Mord. Tat an jungem Russen in Euskirchen ist eine Propaganda-Erfindung. *Frankfurter Allgemeine Zeitung.* <https://www.faz-biblionet.de/faz-portal/document?uid=FAZ__FD12022032250001449368440>.

D106: Balser, M., & Steinke, R. (20 March 2022). Ukraine: Deutsche Extremisten wollen an die Front. *Süddeutsche Zeitung.* <https://www.sueddeutsche.de/politik/ukraine-krieg-rechtsradikale-deutschland-1.5551336?reduced=true>

D107: Fischer, S. (11 July 2021). Podcast "Cui Bono": Lautsprecher. *Süddeutsche Zeitung.* <https://www.sueddeutsche.de/medien/podcast-cui-bono-ken-jebsen-1.5348149?reduced=true>

D108: Eder, S. (7 March 2022). Pandemie, Krieg und Propaganda. *Frankfurter Allgemeine Zeitung*. <https://www.faz-biblionet.de/faz-portal/document?uid=FAZ__FD12022030750001389912998>.

D109: Steinke, R., & Strittmatter, K. (16 March 2022). Russlands Informationskrieg: Am Anfang war die Lüge. *Süddeutsche Zeitung.* <https://www.sueddeutsche.de/politik/russland-hacker-cyberwar-1.5548294?reduced=true>

D110: Lange, D. (2021). *RT Deutsch INSIDE. Putins Medienarmee in Deutschland.* Independently published.

D111: kla.tv. (31 July 2016). Friedensfahrt Berlin-Moskau 7.-21.8.2016 – Interview mit Initiant Dr. Rainer Rothfuß. *kla.tv.* <https://www.kla.tv/8742>

D112: Agence France-Presse. (28 May 2022). Russische Stiftung soll rechtsextreme Szene in Deutschland finanziell unterstützen. *Die Welt.* <https://www.welt.de/politik/deutschland/article239040647/Russische-Stiftung-soll-rechtsextreme-Szene-in-Deutschland-finanziell-unterstuetzen.html>

D113: n.a. (22 October 2020). ІХ МЕЖДУНАРОДНАЯ КОНФЕРЕНЦИЯ. *РУССКОЕ ПОЛЕ.* <http://www.russkoepole.it/ix-mezhdunarodnaya-konferencziya/>

D114: TASS. (25 September 2022). International observers note voluntarily nature of referendums in Donbass. *TASS Russian News Agency.* <https://tass.com/world/1513167>

D115: n.a. (n.d.). Baab, Patrik. *NachDenkSeiten.* <https://www.nachdenkseiten.de/?tag=baab-patrik>

D116: Wienand, L. (27 September 2022). Deutsche Helfer in der Ostukraine: Scheinreferendum, hurra! *t-online.* <https://www.t-online.de/nachrichten/ukraine/id_100057900/noch-mehr-deutsche-beobachter-bei-russlands-schein-referenden-in-der-ukraine-.html>

D117: n.a. (n.d.). 2023 Die Druschba Aktionen und Touren. *Druschba-Global* <https://druschba-global.org/2023-2/>

D118: n.a. (n.d.). 2019 Krim. *Druschba-Global.* <https://druschba-global.org/2019-krim/>

D119: Nikolskaya, P., Saito, M., Tsvetkova, M., & Zverev, A. (3 January 2023). Pro-Putin operatives in Germany work to turn Berlin against Ukraine. *Reuters Investigates*. <https://www.reuters.com/investigates/special-report/ukraine-crisis-germany-influencers/>

D120: Argüeso, O., Bensmann, M., & Joeres, A. (28 February 2022). Russlands Krieg entlarvt Europas Rechte. *Correctiv.* <https://correctiv.org/aktuelles/2022/02/28/russlands-krieg-entlarvt-europas-rechte/>

D121: Kohrs, C. (27 December 2016). Das Zentralorgan der AfD. *Correctiv.* <https://correctiv.org/?p=21792?edit_off>

D122: Kohrs, C. (30 December 2016). Das Böse ist immer und überall. *Correctiv.* <https://correctiv.org/aktuelles/neue-rechte/2016/12/30/das-boese-ist-immer-und-ueberall/>

D123: Bundestag. (24 November 2017). Sachstand: Ausgewählte politisch-publizistische Internetportale. *Deutscher Bundestag.* <https://www.bundestag.de/resource/blob/537216/af898d912097745d27329bc9d3dc13b5/WD-10-054-17-pdf-data.pdf>

D124: Röttger, T., & Timmermann, S. (29 March 2022). Corona-Kritiker bleiben auch im Krieg laut – doch nicht alle schlagen sich auf die Seite Russlands. *Correctiv.* <https://correctiv.org/faktencheck/hintergrund/2022/03/29/corona-kritiker-bleiben-auch-im-krieg-laut-doch-nicht-alle-schlagen-sich-auf-die-seite-russlands/>

D125: Rathje, J., Dittrich, M., & Müller, M. (18 March 2022). Telegram-Analyse zum Ukraine-Krieg: RT DE dominant im verschwörungsideologischen Milieu. *Center für Monitoring, Analyse und Strategie (CeMAS).* <https://cemas.io/blog/telegram-rt-ukraine/>

D126: Rathje, J. (11 March 2022). Verschwörungsideologische Positionierungen zum russischen Angriffskrieg gegen die Ukraine. *Center für Monitoring, Analyse und Strategie (CeMAS).* <https://cemas.io/blog/positionen-ukraine/>

D127: Spahn, S., Vogel, S., Warda, K., & Meisner, M. (March 2022). Monitoring März: Krieg gegen die Ukraine. *Gegneranalyse.* <https://gegneranalyse.de/monitoring-maerz/>

D128: Meisner, M., Vogel, S., Warda, K., & Spahn, S. (April 2022). Monitoring April: Krieg gegen die Ukraine 2. *Gegneranalyse.* <https://gegneranalyse.de/monitoring-april/>

D129: Linden, M. (March 2022). Fallstudie NachDenkSeiten: Vom Aufklärungs- zum Querfront-Medium? *Gegneranalyse.* <https://gegneranalyse.de/fallstudie-1-nachdenkseiten/>

D130: n.a. (n.d.). Mitglieder. *Ludwig-Erhard-Stifung.* <https://www.ludwig-erhard.de/die-stiftung/mitglieder/>

D131: n.a. (n.d.). Institut für Staatspolitik (IfS). *Bundeszentrale für politische Bildung.* <https://www.bpb.de/themen/rechtsextremismus/dossier-rechtsextremismus/500828/institut-fuer-staatspolitik-ifs/>

D132: n.a. (n.d.). Politically Incorrect (PI-News.de). *Bundeszentrale für politische Bildung.* <https://www.bpb.de/themen/rechtsextremismus/dossier-rechtsextremismus/500837/politically-incorrect-pi-news-de/>

D133: Smirnova, J., & Arcostanzo, F. (1 March 2022). German-Language Disinformation about the Russian Invasion of Ukraine on Facebook. *Institute for Strategic Dialogue.* <https://www.isdglobal.org/digital_dispatches/german-language-disinformation-about-the-russian-invasion-of-ukraine-on-facebook/>

D134: Bensmann, M., Eckert, T., & Richter, F. (30 April 2020). „Hygiene-Demos“: Russland-Freunde gegen Corona. *Correctiv.* <https://correctiv.org/aktuelles/2020/04/30/hygiene-demos-russland-freunde-gegen-corona/>

D135: Kohrs, C. (28 December 2016). Der Schirmherr der völkischen Bewegung. *Correctiv.* <https://correctiv.org/aktuelles/neue-rechte/2016/12/28/der-schirmherr-der-voelkischen-bewegung/>

D136: Kohrs, C. (2 January 2017). Am besten hetzt es sich anonym. *Correctiv.* <https://correctiv.org/aktuelles/neue-rechte/2017/01/02/am-besten-hetzt-es-sich-anonym/>

D137: Kohrs, C. (4 January 2017). Russische Propaganda für deutsche Zuschauer. *Correctiv.* <https://correctiv.org/aktuelles/neue-rechte/2017/01/04/russische-propaganda-fuer-deutsche-zuschauer/>

D138: Redaktion Belltower.News. (21 December 2021). Rechtsalternative Medien: Der Verschwörungssender AUF1-TV. *Belltower.News.* <https://www.belltower.news/rechtsalternative-medien-der-verschwoerungssender-auf1-tv-126097/>

D139: Balzer, E. (20 December 2021). Compact-Magazin: Hand in Hand mit Rechtsextremen. *Belltower.News.* <https://www.belltower.news/compact-magazin-hand-in-hand-mit-rechtsextremen-126007/>

D140: Balzer, E. (2 June 2022). Desinformations-Medien: Der Anti-Spiegel – Russische Propaganda und Verschwörungsmythen. *Belltower.News.* <https://www.belltower.news/desinformations-medien-der-anti-spiegel-russische-propaganda-und-verschwoerungsmythen-132357/>

D141: Eckert, T., Helberg, C., & Röttger, T. (24 May 2019). Der Geldgeber: Wie das „Middle East Forum“ die Europäische Rechte finanziert. *Correctiv.* <https://correctiv.org/faktencheck/hintergrund/2019/05/24/der-geldgeber-wie-das-middle-east-forum-die-europaeische-rechte-finanziert/>

D142: Eckert, T., Helberg, C., & Röttger, T. (24 May 2019). Der Verbreiter: „Journalistenwatch“ desinformiert mit Geld und Geschichten aus den USA. *Correctiv.* <https://correctiv.org/faktencheck/hintergrund/2019/05/24/der-verbreiter-journalistenwatch-desinformiert-mit-geld-und-geschichten-aus-den-usa/>

D143: Eckert, T. (21 May 2019). „Erste Konferenz der freien Medien“: Wie die AfD rechte Blogger und Identitäre in den Bundestag einlud. *Correctiv.* <https://correctiv.org/faktencheck/hintergrund/2019/05/21/erste-konferenz-der-freien-medien-wie-die-afd-rechte-blogger-und-identitaere-in-den-bundestag-einlud/>

D144: Ayyadi, A. (5 June 2019). “Alternative” Medien: PI-News – Zentrale für Muslimfeindlichkeit, Desinformation und Hetze. *Belltower.News.* <https://www.belltower.news/alternative-medien-pi-news-zentrale-fuer-muslimfeindlichkeit-desinformation-und-hetze-86329/>

D145: Amtsgericht Stendal. (26 April 2019). Satzung vom Verein für Staatspolitik e.V. *FragDenStaat.* <https://fragdenstaat.de/anfrage/satzung-vom-verein-fur-staatspolitik-ev/>

D146: Bundestag. (5 October 2021). Antwort auf die Kleine Anfrage - Drucksache 19/32513 - Verbindungen, Aktivitäten und Akteurinnen und Akteure der Desiderius-Erasmus-Stiftung. *Deutscher Bundestag.* <https://dip.bundestag.de/drucksache/auf-die-kleine-anfrage-drucksache-19-32513-verbindungen-aktivit%C3%A4ten-und-akteurinnen/258059>

D147: Bundestag. (25 June 2021). Schriftliche Fragen mit den in der Woche vom 21. Juni 2021 eingegangenen Antworten der Bundesregierung. *Deutscher Bundestag.* <https://dip.bundestag.de/drucksache/schriftliche-fragen-mit-den-in-der-woche-vom-21-juni/256023>

D148: Bundestag. (3 November 2020). Antwort auf die Kleine Anfrage - Drucksache 19/23406 - Stellung des Compact Magazins im rechtsextremistischen Spektrum. *Deutscher Bundestag.*<https://dip.bundestag.de/drucksache/auf-die-kleine-anfrage-drucksache-19-23406-stellung-des-compact-magazins/246868>

D149: Bundestag (27 April 2022). Antwort auf die Kleine Anfrage - Drucksache 20/1403 - Auslandsverbindungen rechtsextremer Strukturen wie die "Freien Sachsen". *Deutscher Bundestag.* <https://dip.bundestag.de/drucksache/auf-die-kleine-anfrage-drucksache-20-1403-auslandsverbindungen-rechtsextremer-strukturen-wie/260468>

D150: Bau, M., & Timmermann, S. (15 July 2022). Sexuelle Gewalt im Ukraine-Krieg: Warum Lyudmila Denisova ihren Job verlor – und wie die russische Propaganda das ausnutzt. *Correctiv.* <https://correctiv.org/faktencheck/hintergrund/2022/07/15/sexuelle-gewalt-im-ukraine-krieg-warum-lyudmila-denisova-ihren-job-verlor-und-wie-die-russische-propaganda-das-ausnutzt/>

D151: Eckert, T., Helberg, C., & Röttger, T. (24 May 2019). Der Geschichtenerzähler: Beim „Gatestone Institute“ entstehen Falschmeldungen, die bis nach Deutschland wandern. *Correctiv*. <https://correctiv.org/faktencheck/hintergrund/2019/05/24/der-geschichtenerzaehler-beim-gatestone-institute-entstehen-falschmeldungen-die-bis-nach-deutschland-wandern/>

D152: Röttger, T., Echtermann, A., & Eckert, T. (23 September 2021). Wie österreichische Medien in den deutschen Wahlkampf eingreifen. *Correctiv.* <https://correctiv.org/faktencheck/hintergrund/2021/09/23/wie-report24-wochenblick-auf1-infodirekt-den-wahlkampf-zur-bundestagswahl-mit-desinformation-beeinflussen/>

D153: Röttger, T., Echtermann, A., & Eckert, T. (23 September 2021). Wie österreichische Medien in den deutschen Wahlkampf eingreifen (picture). *Correctiv.* <https://correctiv.org/faktencheck/hintergrund/2021/09/23/wie-report24-wochenblick-auf1-infodirekt-den-wahlkampf-zur-bundestagswahl-mit-desinformation-beeinflussen/> (picture)

D154: Titz, U. (8 June 2022). Desinformationskrieg: Aus dem Osten nichts Neues? *Belltower.News.* <https://www.belltower.news/desinformationskrieg-aus-dem-osten-nichts-neues-132557/>

D155: Waldmüller, V. (20 January 2022). Rubikon News: Ein Querfront-Magazin? *Belltower.News.* <https://www.belltower.news/rubikon-news-ein-querfront-magazin-127171/>

D156: Rafael, S. (15 September 2021). Bundestagswahl 2021: Wie einflussreich sind Desinformationen von “RT DE”? *Belltower.News.* <https://www.belltower.news/bundestagswahl-2021-wie-einflussreich-sind-desinformationen-von-rt-de-121111/>

D157: Lauer, S. (14 June 2017). Wie Geflüchtete die “Epoch Times” gerettet haben – Teil 1. *Belltower.News.* <https://www.belltower.news/wie-gefluechtete-die-epoch-times-gerettet-haben-teil-1-44264/>

D158: Dun & Bradstreet Firmenprofile. (31 December 2021). Firmenprofil Epoch Times. *Dun & Bradstreet Deutschland GmbH.* <https://www.wiso-net.de/document/DUEE__75a580446fe15b1e27801a8373c13b89267825cb>

D159: Meisner, M. (June 2022). Monitoring 08: Anti-Spiegel. *Gegneranalyse.* <https://gegneranalyse.de/monitoring-08-anti-spiegel/>

D160: Spahn, S. (April 2022). Monitoring 06: Neues aus Russland. *Gegneranalyse.* <https://gegneranalyse.de/monitoring-06-neues-aus-russland/>

D161: Marinov, V., & Bau, M. (8 April 2022). Alina Lipp: Wie eine 28-Jährige zum Sprachrohr russischer Propaganda wurde. *Correctiv.* <https://correctiv.org/faktencheck/hintergrund/2022/04/08/alina-lipp-wie-eine-28-jaehrige-zum-sprachrohr-russischer-propaganda-wurde/>

D162: Livshits, A. (22 June 2022). Kettenbriefe für den Kreml: Wie russische Propaganda in Deutschland verfängt. *Belltower.News.* <https://www.belltower.news/kettenbriefe-fuer-den-kreml-wie-russische-propaganda-in-deutschland-verfaengt-133569/>

D163: Amadeu Antonio Stiftung. (2022). COVID-Leugner:innen unterstützen Putins Infokrieg – Deutsche Nazis unterstützen ukrainische Ultranationalisten“. *Amadeu Antonio Stiftung.* <https://www.amadeu-antonio-stiftung.de/wp-content/uploads/2022/03/analyse-papier-russland-ukraine-krieg.pdf>.

D164: Linden, M. (10 May 2022). Claqueure des Angriffskriegs – Putins willige „Alternativmedien“ und ihre politischen Repräsentanten. *Dialog Forum.* <https://forumdialog.eu/2022/05/10/claqueure-des-angriffskriegs-putins-willige-alternativmedien-und-ihre-politischen-repraesentanten/>

D165: Bergholz, A. (24 February 2022). Putins nützliche Idioten: Querdenker, AfD & Co. verharmlosen Krieg in der Ukraine. *Der Volksverpetzer.* <https://www.volksverpetzer.de/social-media/ukraine-krise-afd-quer/>

D166: Segantini, J. (21 June 2022). Kremltreue Propaganda von Alina Lipp & Co.: Die Welt der Putinfluencer. *Der Volksverpetzer.* <https://www.volksverpetzer.de/ukraine/alina-lipp-putinfluencer/>

D167: Livshits, A. (24 March 2022). Krieg gegen die Ukraine: Wieso “Querdenken” zu Putin hält. *Belltower.News.* <https://www.belltower.news/krieg-gegen-die-ukraine-wieso-querdenken-zu-putin-haelt-129531/>

D168: Echtermann, A. (21 February 2020). Gesucht: Influencer*in, jung, rechts. *Correctiv.* <https://correctiv.org/faktencheck/hintergrund/2020/02/21/gesucht-influencerin-jung-rechts/>

D169: Lauer, S. (16 April 2020). Coronavirus-Querfront: Keine Abgrenzung nach rechtsaußen. *Belltower.News.* <https://www.belltower.news/coronavirus-querfront-keine-abgrenzung-nach-rechtsaussen-98345/>

D170: Balzer, E. (12 July 2022). Klagemauer.TV: Eine Rechtsaußen-Sekte und ihre Verschwörungserzählungen. *Belltower.News.* <https://www.belltower.news/klagemauer-tv-eine-rechtaussen-sekte-und-ihre-verschwoerungserzaehlungen-134727/>

D171: Wietlisbach, O. (10 March 2022). Warum «Querdenker» und Impfgegner jetzt pro Putin sind. *watson.* <https://www.watson.ch/digital/international/160379622-ukraine-warum-querdenker-und-impfgegner-nun-putins-luegen-verbreiten>

D172: Linden, M. (May 2022). ‌„Z“ wie End­kampf – Die verschwörungsideologische Renaissance der Apokalypse. *Gegneranalyse.* <https://gegneranalyse.de/markus-linden-z-wie-endkampf-die-renaissance-der-apokalypse/>

D173: Spahn, S. (6 June 2016). Analyse: Das Ukraine-Bild in Deutschland. *Bundeszentrale für politische Bildung.* <https://www.bpb.de/themen/europa/russland-analysen/nr-317/228854/analyse-das-ukraine-bild-in-deutschland/>

D174: Havlicek, S., Applebaum, A., Pomerantsev, P., Smith, M., & Colliver, C. (2017). ‘Make Germany Great Again’: Kremlin, Alt-Right, and International Influences in the 2017 German Elections. *London School of Economics Report*. <https://www.isdglobal.org/isd-publications/make-germany-great-again-kremlin-alt-right-and-international-influences-in-the-2017-german-elections/>

D175: klicksafe. (January 2021). Ethik macht klick - Meinungsbildung in der digitalen Welt. *klicksafe.* <https://www.klicksafe.de/fileadmin/cms/download/Material/P%C3%A4d._Praxis/Lehrer_LH_Ethik_II_Meinungsbildung_MeinungsBildungsKompetenz_RhP.pdf>

D176: ISD. (18 July 2022). Gepampert vom Kreml: Wie westliche Influencer:innen pro-russische Botschaften über den Krieg in der Ukraine verbreiten. *Institute for Strategic Dialogue Germany.* <https://isdgermany.org/gepampert-vom-kreml-wie-westliche-influencerinnen-pro-russische-botschaften-ueber-den-krieg-in-der-ukraine-verbreiten/>

D177: Smirnova, J., & Arcostanzo, F. (1 April 2022). Russia-Ukraine War Sparks Influx of Disinformation in German-language Conspiracy Groups. *Institute for Strategic Dialogue.* <https://www.isdglobal.org/digital_dispatches/russia-ukraine-war-sparks-influx-of-disinformation-in-german-language-conspiracy-groups/>

D178: Smirnova, J., Matlach, P., & Arcostanzo, F. (4 March 2022). Support from the Conspiracy Corner: German-Language Disinformation about the Russian Invasion of Ukraine on Telegram. *Institute for Strategic Dialogue.* <https://www.isdglobal.org/digital_dispatches/support-from-the-conspiracy-corner-german-language-disinformation-about-the-russian-invasion-of-ukraine-on-telegram/>

D179: de:hate. (23 January 2023). Russische Propaganda – Desinformation im Krieg. *Belltower.News.* <https://www.belltower.news/dehate-report-4-russische-propaganda-desinformation-im-krieg-145523/>

D180: Broder, H. M. (7 June 2022). Leider wahr: Die Putin-Trolle gibt es wirklich. *Twitter.* <https://twitter.com/Achgut_com/status/1534175340641374208>

D181: Stein, D. (9 June 2022). Claudia Roth, grüne Kulturstaatsministerin, sorgt sich um die kulturelle Identität der ukrainischen Nation. *Twitter.*  <https://twitter.com/Dieter_Stein/status/1534841012446801920?s=20>

D182: Stein, D. (9 June 2022). Claudia Roth, grüne Kulturstaatsministerin, sorgt sich um die kulturelle Identität der ukrainischen Nation (picture). *Twitter.*  <https://twitter.com/Dieter_Stein/status/1534841012446801920?s=20>

D183: Stein, D. (5 June 2022). Stimme der Vernunft aus der Schweiz. *Twitter.* <https://twitter.com/Dieter_Stein/status/1533474895639764992?s=20>

D184: n.A. (26 September 2022). Foreign “journalists” with ties to RF were among the “international observers” at the sham referendum – list. *Інститут Масової Інформації.* <https://imi.org.ua/en/news/foreign-journalists-with-ties-to-rf-were-among-the-international-observers-at-the-sham-referendum-i47970>

D185: Fischer, K. (11 March 2016). Kasse machen mit den Rechtspopulisten. *WirtschaftsWoche.* <https://www.wiwo.de/politik/deutschland/afd-herr-elsaesser-auf-hass-tournee/13045664-2.html>

D186: Friends of Crimea. (18 December 2021). Alina Lipp, Deutschland - Zur Unterstützung der Krim. *YouTube.* <https://www.youtube.com/watch?v=u16ZRafnt30>

D187: n.a. (n.d.). Wladimir Putin Reden an die Deutschen. *ZVAB.* <https://www.zvab.com/Wladimir-Putin-Reden-Deutschen-Juergen-Elsaesser/22494136926/bd>

D188: Ottov.Freising. (24 June 2014). Putins Reden an die Deutschen (Jürgen Elsässer & Yasmine Pazio). *gloria.tv.* <https://gloria.tv/post/cso8tYC97gw44i2QHSi3ZWF11>

D189: Berger, J. (7 March 2022). NachDenkSeiten-Service: Wie Sie RT und SNA noch erreichen können. *NachDenkSeiten.* <https://www.nachdenkseiten.de/?p=81643>

D190: Röper, T. (19 March 2023). Besuch an der Front nahe Donezk und mehrere Interviews. *Anti-Spiegel.* <https://www.anti-spiegel.ru/2023/besuch-an-der-front-nahe-donezk-und-mehrere-interviews/>

D191: Biegert, S. (10 November 2017). Angela Würtz war mit 300 anderen Deutschen auf Russlandreise. *baden online.* [https://www.bo.de/lokales/achern-oberkirch/angela-wuertz-war-mit-300-anderen-deutschen-auf-russlandreise#](https://www.bo.de/lokales/achern-oberkirch/angela-wuertz-war-mit-300-anderen-deutschen-auf-russlandreise)
